# cepip: context-dependent epigenomic weighting for prioritization of regulatory variants and disease-associated genes

**DOI:** 10.1186/s13059-017-1177-3

**Published:** 2017-03-16

**Authors:** Mulin Jun Li, Miaoxin Li, Zipeng Liu, Bin Yan, Zhicheng Pan, Dandan Huang, Qian Liang, Dingge Ying, Feng Xu, Hongcheng Yao, Panwen Wang, Jean-Pierre A. Kocher, Zhengyuan Xia, Pak Chung Sham, Jun S. Liu, Junwen Wang

**Affiliations:** 1 Department of Pharmacology, School of Basic Medical Sciences, Tianjin Medical University, Tianjin, China; 2 Department of Medical Genetics, Center for Genome Research, Zhongshan School of Medicine, Sun Yat-sen University, Guangzhou, China; 3 Centre for Genomic Sciences, The University of Hong Kong, Hong Kong SAR, China; 4 Department of Anaesthesiology, The University of Hong Kong, Hong Kong SAR, China; 5 School of Biomedical Sciences, The University of Hong Kong, Hong Kong SAR, China; 6 Department of Psychiatry, The University of Hong Kong, Hong Kong SAR, China; 7 Centre for Reproduction, Development and Growth, LKS Faculty of Medicine, The University of Hong Kong, Hong Kong SAR, China; 8 Department of Microbiology, Immunology and Molecular Genetics, University of California, Los Angeles, CA 90095 USA; 9 Department of Statistics, Harvard University, Cambridge, Boston, MA 02138-2901 USA; 10 Department of Health Sciences Research & Center for Individualized Medicine, Mayo Clinic, Scottsdale, AZ 85259 USA; 11 Department of Biomedical Informatics, Arizona State University, Scottsdale, AZ 85259 USA

**Keywords:** Regulatory variant, Variant prioritization, Disease-susceptible gene, Cell type-specific, Epigenome

## Abstract

It remains challenging to predict regulatory variants in particular tissues or cell types due to highly context-specific gene regulation. By connecting large-scale epigenomic profiles to expression quantitative trait loci (eQTLs) in a wide range of human tissues/cell types, we identify critical chromatin features that predict variant regulatory potential. We present cepip, a joint likelihood framework, for estimating a variant’s regulatory probability in a context-dependent manner. Our method exhibits significant GWAS signal enrichment and is superior to existing cell type-specific methods. Furthermore, using phenotypically relevant epigenomes to weight the GWAS single-nucleotide polymorphisms, we improve the statistical power of the gene-based association test.

**Electronic supplementary material:**

The online version of this article (doi:10.1186/s13059-017-1177-3) contains supplementary material, which is available to authorized users.

## Background

Complex traits are usually affected by a large number of genetic factors, jointly contributing to the susceptibility of the disease and the phenotype [1, 2]. Recent genome-wide association studies (GWASs) have successfully identified tens of thousands of significant trait/disease-associated single-nucleotide polymorphisms (SNPs) in humans [3, 4], but these explain only a modest proportion of the heritability [5]. Identifying causal variants with moderate effect size underlying the missing heritability is currently one of the biggest challenges [6, 7]. The majority of GWAS risk loci, as well as loci with sub-genome-wide significance (*P* values between 1 × 10^−5^ and 5 × 10^−8^), localize to non-coding genomic regions with many gene regulatory signals [3], suggesting that most trait/disease causal SNPs exert their phenotypic effects by altering gene expression [8, 9]. This is further supported by GWAS risk loci being enriched in genomic regions with many expression quantitative trait loci (eQTLs) and open chromatins [10–13]. Therefore, accurate identification of functional regulatory variants would facilitate the discovery of novel loci and genes that affect complex traits and diseases.

Genes are regulated in a highly context-specific manner. Both genetic and epigenetic gene regulations are tissue/cell type-specific and depend on chromatin states and interactions [14, 15]. Studies have shown that trait/disease-associated variants are significantly enriched in chromatin states of relevant tissues and cell types [16–19]. These findings indicate that cell type-specific chromatin marks are important for prioritizing putative regulatory variants. Several studies have incorporated cell type-specific genomic/epigenomic annotations into the framework for prioritizing non-coding regulatory variants [20–22] or for fine-mapping GWAS causal variants [23–27]. However, the selection of the most informative chromatin marks and their combined effects underlying the variant’s regulatory potential has not been well studied. In addition, recent human cell population-based or tissue-based eQTL mapping studies, such as the Genotype-Tissue Expression Project (GTEx) [11], have provided an unprecedented opportunity to explore context-dependent regulatory patterns surrounding these loci. Therefore, new computational methods that integrate tissue/cell type-specific eQTL data with coordinated epigenomic profiles are needed to better prioritize regulatory variants and disease-associated genes.

We previously developed an ensemble model to integrate predicted scores from CADD [28], FunSeq [29, 30], GWAVA [31], and GWAS3D [16] to compute the composite likelihood of a given variant affecting the gene regulation [32]. We showed that this model outperformed each individual method using various benchmarks [32]. In this study, we used epigenomic maps of 127 tissues/cell types from the Roadmap Epigenomics Project [33] to develop a context-dependent model that could examine important chromatin features surrounding an eQTL and predict its regulatory potential. We further combined this model with our previous ensemble model to improve the predictions. Using independent eQTL and GWAS benchmarks, we demonstrated our novel approach was superior to existing cell type-specific methods and the predictions showed significant enrichment of genome-wide significant variants identified by GWAS (GWAS signals). The context-dependent combined probability was then incorporated into our previous gene-based association test (GATES) [34] that weighted each GWAS variant. We found this weighting strategy could increase the power of detection of disease-associated genes driven by regulatory variants. The cepip software and the source code are freely available at http://jjwanglab.org/cepip or https://github.com/mulin0424/cepip under the GNU General Public License v3.

## Results

### Exploring chromatin marks around eQTLs identifies critical chromatin features in associated cell types

To illustrate distinct regulatory effects of functional variants in different tissues/cell types, we used a uniformly processed dataset of fine-mapped *cis*-eQTLs from 11 gene expression studies on seven tissues/cell types [35]. The fine-mapped eQTL SNPs in a specific tissue/cell type can be used as indicators to identify regulatory signals of associated loci under certain conditions. For each tissue/cell type-specific eQTL dataset, we sampled the control SNPs using two different matching schemes: (1) by random allele frequency-matched sampling around the transcription start site (TSS); and (2) by strict sampling, taking into consideration potential causal LD, allele frequency, distance to TSS, and GC content around the SNP locus. Using ENCODE cell lines matched to these tissues/cell types (Additional file 1: Table S1), we extracted 36 chromatin features (DNase I hypersensitive sites [DHSs] and histone modification marks) for each of the eQTLs and the two corresponding control SNPs. These features included the intersection of the queried locus with a chromatin mark (Hit), the intensity of an overlapping mark (Intensity), and the distance between the queried locus and the peak summit of a chromatin mark (Centrality).

**Fig. 1:**
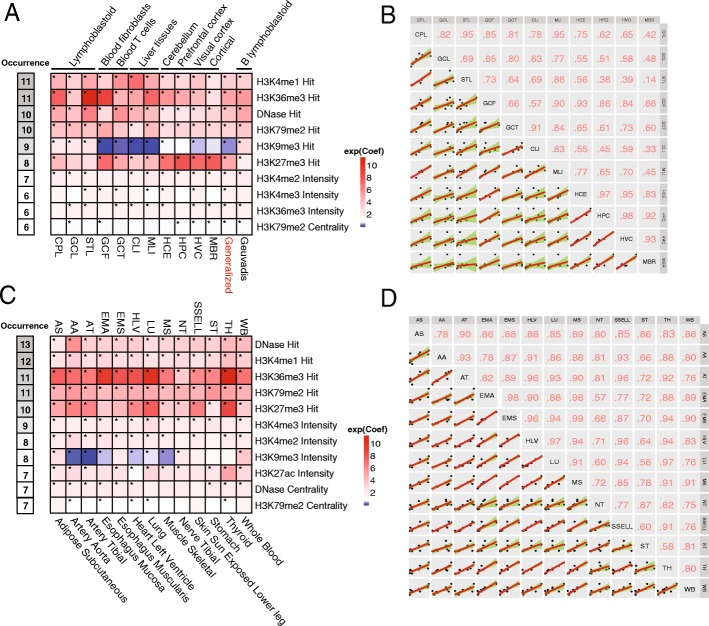
Critical chromatin features and correlations among tissues/cell types. **a** Tissue/cell type-specific or generalized logit models trained by ten selected chromatin features using eQTLs fine mapping dataset (occurrence indicates the number of models sharing the feature after the feature selection procedure; * indicates the *P* value of coefficient for corresponding feature < 0.05; heatmap color is rendered by exponential coefficients). *CPL* CAP_LCL, *STL* Stranger_LCL, *HCE* Harvard_cerebellum, *HPC* Harvard_prefrontal_cortex, *HVC* Harvard_visual_cortex, *GCF* GenCord_fibroblast, *GCL* GenCord_LC, *GCT* GenCord_tcell, *CLI* UChicago_liver, *MLI* Merck_liver, *MBR* Myers_brain, *All* combined dataset, *Geuvadis* Geuvadis_LCL. **b** Spearman’s rank correlation tests between the coefficients of the selected features in each cell type-specific logit model using ten selected chromatin features. **c** Tissue/cell type-specific logit models trained by 11 selected chromatin features using the GTEx eQTLs dataset (occurrence indicates the number of models sharing the feature after the feature selection procedure; * indicates the *P* value of coefficient for corresponding feature < 0.05; heatmap color is rendered by exponential coefficients). **d** Spearman’s rank correlation between the coefficients of selected features in each GTEx cell type-specific logit model using 11 selected chromatin features

For each of the 11 eQTL studies, we trained the logit model to estimate the probability that the 36 chromatin features were associated with eQTLs (herein referred to as the “regulatory potential” of the observed variants), which resulted in 11 models. To identify the most informative feature set, we used a backward stepwise regression based on the Akaike Information Criterion (AIC) [36] to select key features, which reduced the number to 9–14 features. Next, we reviewed the contribution of each feature in terms of its importance and consistency. We found that seven or more features were shared by more than half of the 11 models using different controls and the “Hit” features were more important than the “Intensity” and “Centrality” features (Fig. 1a, also Additional file 1: Figure S1A, Tables S2 and S3). We termed the features with high occurrences (appearing in more than half of the models) as “selected chromatin features.” Among these selected chromatin features, H3K4me1 Hit, H3K36me3 Hit, DHS Hit, and H3K79me2 Hit were present in most of the models.

These highly reoccurring chromatin signals included some well-established chromatin marks such as enhancer marks (H3K4me1), active gene bodies (H3K36me3), active promoters, and enhancers or transcribed regions (DHS and H3K79me2). Additionally, H3K9me3 Hit/Intensity and H3K4me3 Intensity were selected features, although H3K9me3 showed inconsistent contributions among different tissue/cell type-specific models. Another repressive mark H3K27me3 Hit was also a selected feature in the random allele frequency-matched sampling models, indicating some eQTLs could modulate gene silencing. Other frequent features, such as H3K36me3 Intensity and H3K79me2 Centrality in the random allele frequency-matched sampling models, could be used to distinguish two variants located within the same ChIP-seq peak (Fig. 1a). Furthermore, H4K20me1 Hit was identified in strict control models. Since this mark has been reported to correlate with high CpG promoters, it suggests that models trained by strict control may capture signals for CpG-dependent promoters (Additional file 1: Figure S1A). Taken together, these results support the previous findings that certain cell type-specific chromatin marks can be predictive of causal regulatory variants [17, 18, 33].

Previous studies reported that H3K27ac was associated with many disease-related variants [33]. However, this active enhancer mark was not selected in most of our tissue/cell type-specific models. We examined the relationship between DHS-related and histone modification features and found that DHS Hit and H3K27ac Hit had the highest correlation among all examined pairs (*r* = 0.542, *P* value < 2.2 × 10^−16^, Pearson’s correlation). To investigate whether additional histone modification features could be selected in the absence of DHS-related features, we retrained our models without DHS-related features. This resulted in H3K27ac Hit appearing in more than half of the models, which implies that it is a critical chromatin feature. In addition, occurrences of H3K4me2 Intensity and H3K4me3 Intensity increased in all cell type-specific models (Additional file 1: Figure S1B, C, Tables S4 and S5). These results indicate DHS-related features could cover signals from some histone modification features in open chromatin regions.

The GTEx project has provided us with eQTLs mapping of many different human tissues. To determine whether the chromatin features identified above are also critical in GTEx data, we used our feature selection procedure to examine eQTLs from 13 human tissues with the epigenomic profiles of relevant tissues/cell types from Roadmap Epigenomics Project (Additional file 1: Table S6). As expected, we found that the majority of previously selected chromatin features were also picked up. Similar levels of occurrence for most of the informative features, such as DHS Hit, H3K4me1 Hit, H3K36me3 Hit, and H3K79me2 Hit, were identified. H3K9me3 Hit was replaced by H3K9me3 Intensity in the list of the selected features, whereas two other features, H3K27ac Intensity and DHS Centrality, were added to the list (Fig. 1c and Additional file 1: Table S7). These results show that several critical chromatin marks display consistent patterns associated with locus regulatory potential under different cellular conditions.

To investigate the effect of the selected chromatin features on predicting regulatory potential across different tissues/cell types, we retrained the logit model using the selected features from the 11 eQTL studies and 13 GTEx eQTL studies, respectively. We performed pairwise correlation tests on the coefficients of the selected features between each of tissue/cell type-specific models. High and consistent correlations among most of the models (Fig. 1b and d) were observed, and the correlations were higher in cell types from the same tissues. These results suggest that our selected features contributed in a generally consistent manner in each context-dependent model and indicate the feasibility of a generalized model.

To improve the model’s predictive power in more conditions, we trained four generalized context-dependent models using the pooled cell type-specific features from all 11 eQTL studies under different controls and related DHS features. Using Geuvadis “the best eQTLs” [37] and epigenomic annotations of GM12878 lymphoblastoid cell line as the gold standard, we found that the generalized context-dependent model trained by random TSS controls and related DHS features slightly outperformed other models (Additional file 1: Figure S2). All selected chromatin features achieved significant coefficients in this generalized model (Additional file 1: Table S8). Also, the coefficients of the newly trained model for Geuvadis data showed a highly correlated pattern with that of the generalized models (Fig. 1a). We, therefore, used this generalized context-dependent model to perform a downstream analysis. Overall, our results highlight that the generalized context-dependent model could consistently predict variant regulatory potential with high accuracy using the ensemble effects of the selected chromatin features.

### Context-dependent scoring of GWAS fine-mapped SNPs underlies phenotypic cell-type specificity

To examine whether our generalized context-dependent model could identify cell-type specificity in human diseases, we first applied our model to 7747 candidate causal SNPs of 38 immune and non-immune diseases/traits derived from a GWAS fine-mapping study [19]. We used reference human epigenomes for 127 diverse tissues/cell types [33] (Additional file 2) to extract the selected chromatin features. We then calculated the regulatory potential for every causal variant in each tissue/cell type.

**Fig. 2:**
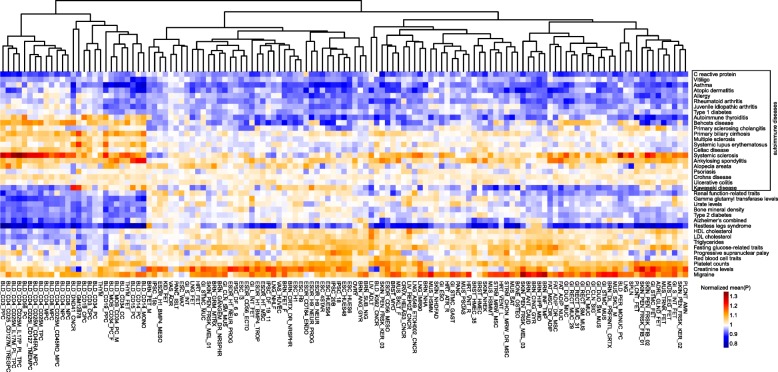
Clustering of 127 tissues/cell types using the normalized mean regulatory potential for fine-mapped GWAS SNPs of 38 immune and non-immune diseases/traits. See Additional file 2 for abbreviations of tissues/cell types

We clustered the 127 tissues/cell types using the normalized mean regulatory potential for all causal SNPs in each disease/trait. The hierarchical clustering generally recapitulated cell lineages. For example, blood cells formed a coherent group, meanwhile brain cells and embryonic stem cells were mostly clustered in separate groups (Fig. 2). Importantly, using our prediction method, we observed phenotypic cell-type specificity for many diseases/traits. The majority of the autoimmune diseases preferentially showed higher mean regulatory potentials for cells within T-cell, B-cell, or monocyte subpopulations (Fig. 2). By ranking the normalized mean regulatory potentials among the 127 tissues/cell types, we estimated the most relevant tissue/cell type in each of the 38 immune and non-immune diseases/traits (Additional file 1: Table S9). As expected, almost all of the autoimmune diseases were successfully mapped to blood-derived cells. Notably, asthma, atopic dermatitis and allergy were mapped to CD4+ T helper cells, whereas alopecia areata and juvenile idiopathic arthritis were mapped to CD4+ CD25+ regulatory T cells. For non-immune diseases, HDL cholesterol, LDL cholesterol, and triglycerides were mapped to liver tissue/cells. Alzheimer’s disease and restless legs syndrome were mapped to brain tissue. These findings were largely in agreement with a recent report showing that H3K27ac enrichment depicts phenotypic cell-type specificity using the same set of causal variants set [19]. Taken together, the integrative effect of our selected chromatin features underlies phenotypic tissue/cell type specificity of the GWAS risk loci in a particular disease/trait.

**Fig. 3:**
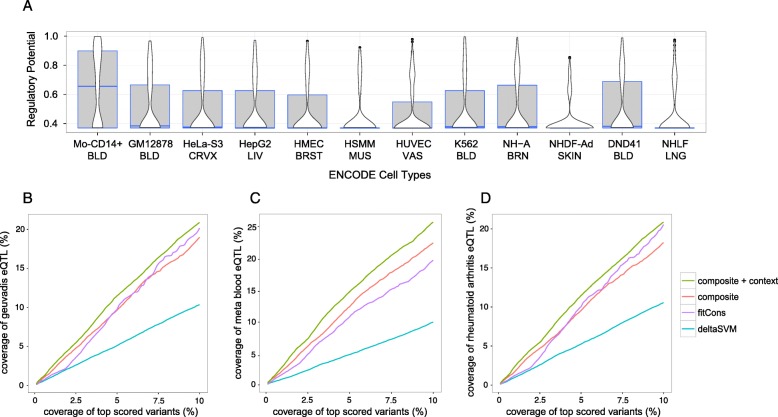
Evaluation of the generalized context-dependent model and combined model. **a** *Boxplot* of cell type-specific regulatory potentials for ImmVar 201 traits/diseases-associated eQTLs using 12 ENCODE cell lines. **b** Coverage of Geuvadis eQTLs with increasing top-ranked variants for three cell type-specific scores and the composite score. **c** Coverage of meta blood eQTLs with increasing top-ranked variants for three cell type-specific scores and composite score. **d** Coverage of RA eQTLs with increasing top-ranked variants for three cell type-specific scores and the composite score

To further test our context-dependent model, we applied it to 201 trait/disease-associated eQTLs from monocytes and/or T cells reported by the ImmVar project [38]. We calculated the regulatory potentials for ImmVar trait/disease-associated eQTLs using epigenomic data of 12 human cell lines from the ENCODE project. It was observed that the regulatory potentials of the 201 eQTLs were significantly higher in Mo-CD14+ cells than in other cell types (*P* < 2.0 × 10^−16^ for all, Mann–Whitney *U* test) (Fig. 3a). This shows our generalized context-dependent model could predict regulatory variants using phenotypic cell type-specific chromatin signatures.

### Cell type-specific evaluation of the combined model

To further enhance the predictive performance, we combined our context-dependent model with a previously reported composite model [32], which can make ensemble predictions from tools such as CADD, FunSeq2, GWAVA, etc. The Pearson correlation coefficient between the regulatory potential and our previous composite probability in the GM12878 cell line at the genome-wide level showed a low dependence (*r* = 0.197, Additional file 1: Figure S3). Assuming independence between the prediction scores from the composite model and the cell type-specific chromatin features, we computed the posterior probability of a variant being regulatory given the two sets of information (herein referred to as “combined probability”). We evaluated the performance of our combined model by comparing it with two recent cell type-specific methods, the evolution-based fitCons [20] and the sequence-based deltaSVM [21], using two independent *cis*-eQTL datasets of relevant tissues/cells (i.e. “Geuvadis eQTLs” from Geuvadis lymphoblastoid cells and “meta blood eQTLs” from an eQTL meta-analysis in non-transformed peripheral blood samples) and a rheumatoid arthritis (RA) eQTL dataset [39].

Assuming an algorithm that covers more eQTLs in top-ranked variants will have better sensitivity in identifying functional regulatory loci, we next investigated the eQTL coverage (excluding eQTLs used in the training dataset) as the percentage of top-ranked variants increased by using all variants from the 1000 Genomes Project. Our combined model can detect more eQTLs in the three independent eQTL datasets than fitCons and deltaSVM among the 10% top-ranked variants. For example, our model and fitCons achieved over 20% coverage in the Geuvadis eQTLs for the 10% top-ranked variants, whereas deltaSVM only achieved 10% coverage (Fig. 3b). Compared with fitCons, our combined model achieved better coverage of the top-ranked variants (Fig. 3b). In addition, our model achieved 26% coverage for the 10% top-ranked variants for meta blood eQTLs, surpassing both fitCons and deltaSVM (19.8% and 10.1% coverage, respectively) (Fig. 3c). Finally, our model achieved 20.8% coverage for the 10% top-ranked variants when applied to RA *cis*-eQTLs, performing better than fitCons and deltaSVM (20.5% and 10.5% coverage, respectively) (Fig. 3d). Our combined model consistently outperformed the previous context-free composite scores in all three evaluations (18.9% for Geuvadis eQTLs, 22.6% for meta blood eQTLs, and 18.3% for RA eQTLs), suggesting that tissue/cell type-specific epigenomic features largely contributed to the combined model (Fig. 3b–d).

**Fig. 4:**
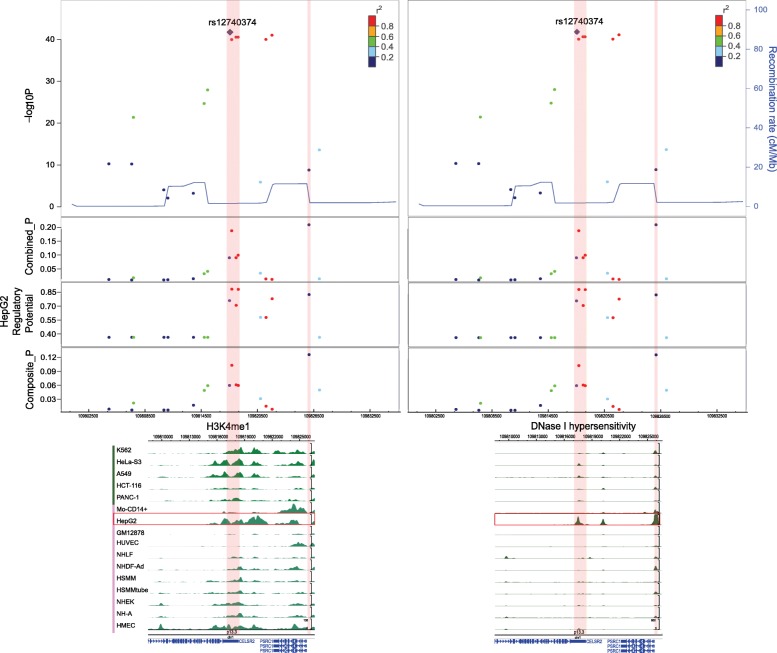
Epigenomic profiles of 1p13.3 vary greatly in different cell lines. Critical chromatin features (H3K4me1 and DHS) around LDL-C-associated SNPs in 1p13.3 (rs12740374 indexed) show large differences among the 16 ENCODE cell lines and specific enrichment in HepG2 cell lines

Next, we used a well-studied GWAS locus in plasma low-density lipoprotein cholesterol (LDL-C) [40] to demonstrate the effectiveness of our method. Based on the above findings (Fig. 2), SNPs associated with multiple lipid metabolism traits were predicted to have higher regulatory potentials in liver cells. We, therefore, selected phenotypically relevant HepG2 epigenomes to prioritize 17 fine-mapped LDL-C-associated SNPs from the 1p13.3 region in Caucasian populations. The regulatory potentials of these 17 SNPs partially correlated with the original GWAS *P* value (*r* = 0.58, *P* value < 0.01, Spearman’s rank correlation) (Additional file 1: Table S10). By checking the epigenomic profiles of HepG2, the top associated SNPs were found to be located in the dips or peaks of H3K4me1 overlapping with DHS (Fig. 4). In addition, the chromatin signals in HepG2 were significantly stronger compared to other ENCODE cell types (Fig. 4), which suggest the importance of cell type-specific prioritization.

Among the 17 statistically fine-mapped LDL-C-associated SNPs, six SNPs were located in an extremely high LD region and had similar *P* values calculated from association studies in a Caucasian population. Thus, it is more challenging to pinpoint true functional SNPs in the Caucasian population with strict LD compared with the African population with its relatively unconfined LD (Additional file 1: Figure S4). Our cell type-specific prioritization, from the perspective of functional gene regulation, can partially resolve this problem by assigning higher regulatory potentials to four SNPs (rs660240, rs646776, rs629301, and rs12740374) (Fig. 4). In addition, the experimentally validated SNP rs12740374 that modulates LDL-C [40] was among the top five variants in our final prioritization list based on either regulatory potential or combined probability (Additional file 1: Table S10). Evidence from the literature and from functional studies support the potential regulatory roles of these top-ranked SNPs (Additional file 1: Table S11), including: (1) HepG2-specific super enhancer or enhancer stretch overlapping with a region containing rs12740374, rs660240, rs629301, and rs646776 [41, 42]; (2) in addition to the experimentally confirmed gene *SORT1* that is regulated by rs12740374, the data from eQTLs and chromosomal long-range interactions revealed more associations of these top-ranked SNPs and other gene targets in different tissues/cell types [43] (Additional file 1: Figure S5); and (3) in silico motif analysis showed altered transcription factor binding affinities with these SNPs [16]. These investigations suggest our method can capture context-dependent regulatory variants in a wide range of gene regulation patterns.

### Top predicted SNPs show higher enrichment of GWAS signals in relevant cells

**Fig. 5:**
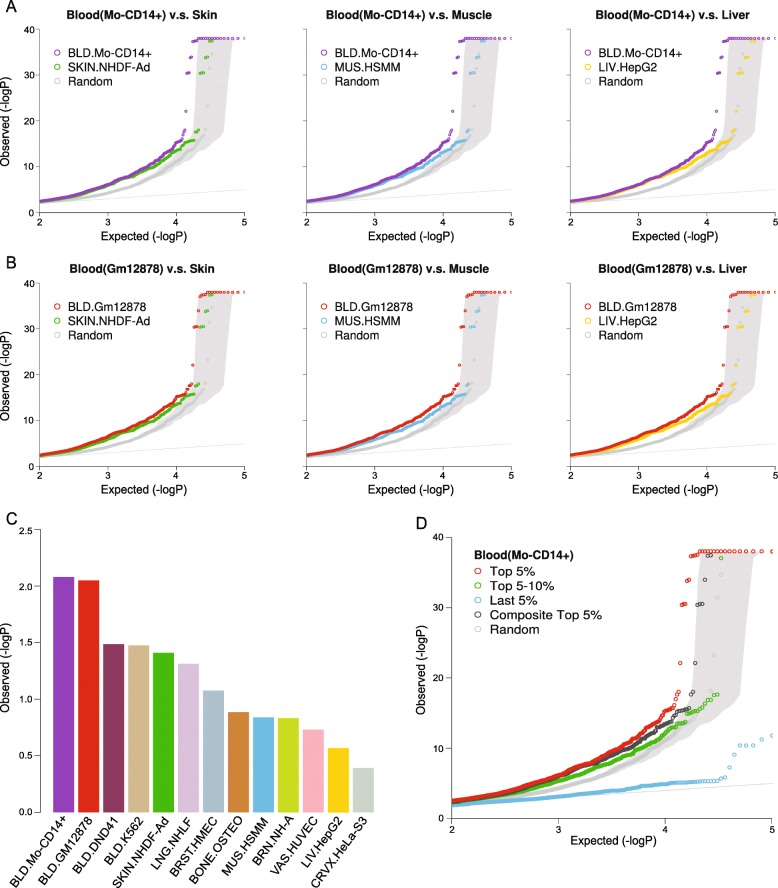
Context-dependent prioritization shows GWAS signal enrichment in relevant cells. **a** The top 5% prioritized SNPs using blood cell line Mo-CD14+ (*purple*) display more leftward shift than using skin (*green*), muscle (*blue*), or liver (*gold*) cell lines against permutated GWAS signals; *gray* area shows 95% intervals of permutated signals. **b** The top 5% prioritized SNPs using blood cell line GM12878 (*red*) display more leftward shift than using skin (*green*), muscle (*blue*), or liver (*gold*) cell lines against permutated GWAS signals; *gray* area shows 95% intervals of permutated signals. **c** The empirical *P* values of permutations for blood cell lines (Mo-CD14+ and GM12878) are more significant than other tissue/cell types. **d** After the blood cell line Mo-CD14+ prioritization, the top-ranked SNPs display more significant shift from permutated GWAS signals than lower-ranked ones; *gray* area shows 95% intervals of permutated signals. *BLD* blood, *LNG* lung, *SKIN* skin, *BONE* bone, *LIV* liver, *VAS* vascular, *MUS* muscle, *BRN* brain, *CRVX* cervix, *BRST* breast, *Composite* context-free composite model

To evaluate the potential of our context-dependent prioritization in genetic mapping studies of diseases, we calculated the combined probability in all 8,253,617 SNPs from a RA GWAS meta-analysis using epigenomic profiles of 13 ENCODE tissues/cell types [44]. Using features from blood cell lines (Mo-CD14+ and GM12878), the top 5% SNPs showed leftward deviations of the observed *P* values from the expected *P* values in the Q-Q plots (Fig. 5a, b). In contrast to other non-blood cell lines (e.g. skin, muscle and liver cell lines), blood-related cells shifted leftward the most (Fig. 5a, b and Additional file 1: Figure S6) and had more significant empirical *P* values by permutation test (Fig. 5c). We observed similar results using allele frequency-matched sampling (Additional file 1: Figure S7). We also calculated the inflation factor (λ) of the top 5% SNPs and found that blood-related cells had higher λ than non-blood cells (Additional file 1: Figure S8). Furthermore, we observed that in the Mo-CD14+ cells, the top-ranked SNPs (top 5%) exhibited more enrichment of RA GWAS signals than lower-ranked SNPs (e.g. top 5–10% and last 5%, Fig. 5d). Similar results were observed in other cell lines (Additional file 1: Figure S9).

To validate the ability of our method to identify regulatory SNPs with moderate GWAS signals (i.e. GWAS *P* values > 5 × 10^–8^), we removed all known RA-associated SNPs and still found similar patterns (Additional file 1: Figure S10). Notably, using epigenomic marks from the Mo-CD14+ cell line, we identified one SNP rs874628 with moderate RA GWAS signals (GWAS *P* value = 0.00036) located in a genomic region enriched with Mo-CD14+ specific chromatin features (Additional file 1: Figure S11). Interestingly, this SNP was reported to be associated with multiple sclerosis (GWAS *P* value = 1.0 × 10^–8^) [17]. We also generated a list of top-prioritized SNPs with moderate RA GWAS *P* values as potential novel candidates for further validation (Additional file 1: Table S12).

To test whether the context-dependent prioritization was affected by LD, we recalculated the ratio of SNPs with sub-genome-wide associations (*P* values < 1 × 10^–5^) by using the effective numbers of independent markers [45]. Using RA-relevant cell types, the combined model significantly increased the ratio compared with the original GWAS signals (Additional file 1: Figure S12 and Table S13). All these results demonstrate the reliability of the context-specific prioritization and its ability to establish a link between variants and a disease.

### Context-dependent epigenomic weighting increases the statistical power of detecting disease-associated genes

We examined if we could improve the statistical power of detecting disease-associated genes driven by regulatory variants by using the context-dependent combined probability as a weight for each GWAS SNP. We incorporated this weighting approach into our previous gene-based association test GATES [34] and then applied it to two RA GWASs [44, 46].

**Fig. 6:**
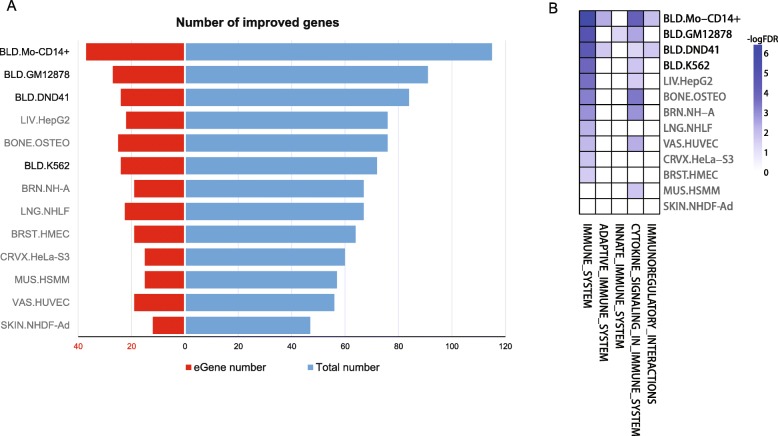
Context-dependent epigenomic weighting improves the detection of disease-associated genes. **a** Number of improved genes by the W_SNP-based gene association test relative to the NW_SNP-based gene association test. *Blue bars* represent the number of all improved genes; *red bars* represent the number of eGenes among the improved genes. **b** Immune system-related pathway enrichment analysis. Blood cell lines are colored in *black* and non-blood cell lines are colored in *gray. BLD* blood, *LNG* lung, *SKIN* skin, *BONE* bone, *LIV* liver, *VAS* vascular, *MUS* muscle, *BRN* brain, *CRVX* cervix, *BRST* breast

For SNPs from the RA GWAS meta-analysis [44], we used the combined probability of 13 different ENCODE cell types as the weight to generate two SNP sets for each cell type: the original set of SNPs with no weights (NW_SNP) and the same set of SNPs with context-dependent weights (W_SNP). Next, we used GATES to examine the RA-associated genes in the NW_SNP and W_SNP sets, respectively. Compared with the GATES detection using the NW_SNP set, we found the W_SNP set improved the power of detection of RA-associated genes in a context-dependent manner. Weighting SNPs with the two blood cell lines (Mo-CD14+ and GM12878) gave the largest number of improved genes as demonstrated by decreasing *P* values (below the significant threshold of 1 × 10^−5^) or by further lowering of the originally significant *P* values to more significant levels (Fig. 6a). It is also noted that using weights from Mo-CD14+ generated more RA eQTL-associated genes (eGenes) [39] when compared with using weights from other cell types (Fig. 6a). This suggests the context-dependent weighting strategy could identify more genes that subsequently affect disease susceptibility through regulatory mechanisms (Additional file 3).

Gene set enrichment analysis revealed that the improved genes weighted by blood cell types showed higher enrichment in immune system pathways than when weighted by other cell types (Fig. 6b and Additional file 1: Table S14). Although some genes became less significant after weighting (worsened genes), the comparisons showed that weighting by blood cell types generated higher percentages of improved genes and lower percentages of worsened genes. Similar results were observed for eGenes (Additional file 1: Figure S13). We performed the same weighting strategy on the other RA study that had a smaller sample size [46]. It is noticed that the improved genes weighted by blood cell lines in this smaller sample size study tended to be genes with higher significance (i.e. smaller *P* values) in the larger sample size study (Additional file 1: Figure S14). Both analyses indicate that our context-dependent epigenomic weighting approach increases the statistical power for the detection of disease-associated genes.

## Discussion

Several computational methods have been developed to predict and prioritize non-coding variants based on only functional annotations, but the performance of these tools have been largely inconsistent [32]. With recent eQTL studies in diverse human tissues/cell types and the availability of large-scale functional genomic data, it is now becoming possible to integrate tissue/cell type-specific chromatin states for predicting regulatory variants and for fine-mapping of disease-causal variants/genes that have weak associations with complex traits [6, 47, 48].

In this study, we describe a novel method to integrate epigenomic features with eQTL data for prioritizing regulatory variants. We uncovered a set of critical chromatin features that could be used to consistently predict variant regulatory potential across different cellular contexts. Building on these findings, we developed a context-dependent combined model to predict variant regulatory potential. By comparing our model with existing methods using multiple functional datasets, we showed that this combined approach greatly improved our original method for identifying regulatory variants and disease-associated genes.

To investigate the relationship between informative tissue/cell type-specific chromatin marks and the regulatory effect of variants, we selected the most significant fine-mapped eQTLs in each LD to form the benchmark datasets. However, these datasets may lose other independent eQTLs within the same LD or introduce false positives, because fine-mapped eQTLs are not necessarily causal ones in the high LD proxies. Nevertheless, due to the high cost of massively parallel experimental validation in real cellular contexts [49–51], fine-mapped eQTL data could be used as a reasonable replacement for a true benchmark dataset. We attempted to represent the regulatory potential of genetic variants by using cell type-specific chromatin states around an eQTL. Using multiple uniformly processed eQTL datasets as benchmarks, our selected chromatin features were found to be generally consistent among different tissues/cell types. These features could be integrated as a weight for GWAS SNPs for prioritizing disease-associated genes. In addition, eQTLs are not enough to explain all functional mechanisms of regulatory variants. We anticipate more large-scale QTL studies focusing on other molecular phenotypes, such as DNA methylation and histone modification, could be available in a wide range of tissues/cell types [27, 52–54].

Genetic determinations of many complex traits are complicated, likely due to the joint susceptibilities of many risk loci with small genetic effect sizes, as well as indirect alterations of protein functions through different levels of gene regulation [26]. Currently, it is difficult to identify true causal variants/genes that underlie disease pathogenesis using conventional genetic mapping strategies alone, because of limitations of sample size, statistical power and LD [47, 55]. Using relevant epigenomic data that match GWAS diseases/traits, we can weigh the regulatory potential of each GWAS SNP and re-evaluate its association independently from LD. Since our method does not rely on GWAS results, the power of detecting causal variants in a particular disease/trait is limited. Nevertheless, the strength of our method is to prioritize active regulatory variants in a certain tissue/cellular environment. Many of our top-prioritized variants, as shown in the results section, revealed strong GWAS signals. In our gene-based association tests, we demonstrated context-dependent epigenomic weighting, which together with the original GWAS summary statistics could boost the detection power of RA-associated genes.

## Conclusions

In summary, we have focused on an essential problem in the field of regulatory variant prioritization and disease-associated gene detection. Considering the importance of cellular chromatin states, we have developed a context-dependent method to quantify the regulatory potential of genetic variants in a particular tissue/cell type. Previous studies suggest that a single tissue/cell type-specific epigenetic mark, such H3K4me3 [18] or H3K27ac [19], could be used to fine-map GWAS loci for particular diseases/traits. Our context-dependent prioritization method uses the integrative effect of multiple chromatin states to identify functional regulatory variants. Building on our previous context-free regulatory variant prediction method, we have demonstrated that context-dependent epigenomic weighting can improve identification of both variant-level and gene-level susceptible loci in GWAS. We will frequently update epigenomes data for more tissues/cell types and integrate cepip into our comprehensive downstream analysis platform KGGSeq in the future [56, 57].

## Methods

### eQTL fine-mapping data and controls

We used the uniformly processed *cis*-eQTLs fine-mapping data produced by Brown et al. [35]. A multi-traits Bayesian linear regression model was used to identify *cis*-eQTLs from 11 studies on seven tissues/cell lines, including CAP_LCL (CPL), Stranger_LCL (STL), Harvard_cerebellum (HCE), Harvard_prefrontal_cortex (HPC), Harvard_visual_cortex (HVC), GenCord_fibroblast (GCF), GenCord_LCL (GCL), GenCord_tcell (GCT), UChicago_liver (CLI), Merck_liver (MLI), and Myers_brain (MBR). We downloaded the eQTL SNPs of the most highly associated *cis*-linked SNP within an LD block. To acquire reliable fine-mapped eQTL SNPs, we applied a strict cutoff of 10% false discovery rate (FDR) for each tissue/cell type. These eQTL SNPs were treated as the candidate regulatory variants. We constructed corresponding background SNPs using two strategies: (1) random allele frequency-matched sampling (less than 0.05 deviations to each eQTL SNP using 1000 Genomes project EUR population) of SNPs around the gene’s nearest TSS genome-wide (within 10 kb); and (2) a strict matching scheme with (i) no overlap with any SNPs within high LD of each fine-mapped eQTL SNP (*r* ^*2*^ > 0.8 in 1000 Genomes project EUR population), (ii) matched allele frequency, (iii) matched TSS distance to each eQTL SNP (up/downstream 1000 bp deviations), and (iv) matched GC content to each eQTL SNP locus (up/downstream 50 bp DNA sequence).

### GTEx eQTL data

We downloaded significant SNP-gene associations from GTEx V4 from the GTEx portal (http://www.gtexportal.org/), which contains Matrix eQTL [58] mapping results of 13 human tissues, including Adipose Subcutaneous (AS), Artery Aorta (AA), Artery Tibial (AT), Esophagus Mucosa (EMA), Esophagus Muscularis (EMS), Heart Left Ventricle (HLV), Lung (LU), Muscle Skeletal (MS), Nerve Tibial (NT), Skin Sun Exposed Lower leg (SSELL), Stomach (ST), Thyroid (TH) and Whole Blood (WB). For each tissue, we sampled equal numbers of frequency-matched background SNPs around the gene’s nearest TSS genome-wide (within 10 kb).

### Functional epigenomics data

We incorporated epigenomic data of 127 human tissues/cell lines from Roadmap Epigenomics Mapping repositories (http://egg2.wustl.edu/roadmap/web_portal/), including primary tissues, diverse blood cells, and embryonic stem cells, as well as ENCODE cell lines. For each tissue/cell line, we acquired narrow peaks using DNase-seq and ChIP-seq for 11 histone modification marks, including H2AFZ, H3K27ac, H3K27me3, H3K36me3, H3K4me1, H3K4me2, H3K4me3, H3K79me2, H3K9ac, H3K9me3, and H4K20me1 from the uniformly reprocessed consolidated epigenome, which can capture relatively equalized signal strength across tissues/cell types. We used imputed peak calls for missing epigenomes [33].

### Cell-type matching and eQTL chromatin signature localization

The 11 eQTL tissues/cell lines and 13 GTEx eQTL tissues were mapped onto the 127 tissues/cell types from Roadmap Epigenomics Project (including 16 ENCODE cell lines) according to their best matched cell type or tissue origin. For eQTL SNPs in each tissue/cell line, we defined three types of feature for the DNase I hypersensitive site and the 11 histone marks using the narrow peak calling results: (1) the hit of peak (Hit) feature; (2) the peak intensity (Intensity) feature; and (3) the distance to summit (Centrality) feature. Overall, we extracted 36 cell type-specific genomic and epigenomic features from all eQTL SNPs and corresponding background SNPs.

### Context-dependent model and model selection

We trained logit model for eQTL SNPs and control SNPs from each of the 11 eQTL studies and calculated the regulatory probability of each investigated SNP. We termed this probability the “regulatory potential” given by:

$$ P\left( causal\Big| X\right)\kern0.5em =\kern0.75em \frac{1}{1\kern0.5em  + {e}^{-\left(\alpha + \beta X\right)}} $$

where α is the intercept and β is the vector of coefficients of *X* (36 features) from the logistic regression of eQTL SNPs of a specific tissue/cell type and corresponding control SNPs. To identify the most informative features, we performed a model selection using backward stepwise selection based on the AIC [59]. We selected features shared by more than half of the tissue/cell type-specific models. Next, we computed the pairwise correlation coefficients for these features using Spearman’s rank correlation tests between models. To make a generalized model, we pooled the cell type-specific chromatin features for each of 11 fine-mapped eQTL datasets and retrained four generalized logit models under different controls and DHS-related features. Four controls include: (1) random control; (2) strict control; (3) random control without DNA-related features; and (4) strict control without DNA-related features. We used Geuvadis and a randomly sampled control around TSS (within 10 kb) to test the generalized models. In addition, the epigenomic annotations of the ENCODE GM12878 lymphoblastoid cell line were used to extract chromatin features.

### Phenotypic cell type-specific evaluation on GWAS fine-mapped data and 127 reference epigenomes

We retrieved candidate causal SNPs from a GWAS fine-mapping study of 38 immune and non-immune diseases (http://www.broadinstitute.org/pubs/finemapping/), which contained 8741 fine-mapped SNPs that passed the PICS probability cutoff (>0.0275). We extracted our selected chromatin features for each candidate regulatory SNPs in the 127 epigenomes. Next, we applied our generalized context-dependent model to predict the regulatory potential for each fine-mapped SNP *k* in the corresponding disease category *i* using each of the consolidated epigenomes of 127 tissues/cell types *j*, defined as *P*_k,i,j_. For each disease and cell/tissue type combination, we calculated the mean prediction score for all causal SNPs that were associated with the disease, defined as the mean_i,j_(*P*). We normalized the mean_i,j_(*P*) across diseases/traits (centered by 1) and used hierarchical clustering to group the tissues/cell types according to the normalized value. We estimated the most relevant tissue/cell type for each of the 38 immune and non-immune diseases/traits by selecting the largest normalized mean_i,j_(*P*) among the 127 tissues/cell types.

### Evaluation of ImmVar eQTLs

We collected an independent dataset of ImmVar eQTLs of the GWAS LD proxy for monocytes and/or T cells to test the performance of our generalized context-dependent model. We calculated the regulatory potential for ImmVar trait/disease-associated eQTL SNPs using the chromatin signatures of 12 ENCODE cell types with complete epigenome profiles (four blood cell lines and eight other cell lines from unique tissues). The Mann–Whitney *U* test was used to compare the regulatory potentials between different measurements after removing SNPs that had the same probability in all cell types.

### Combined model

We used the ensemble model [60] and our context-dependent model in a combined model, integrating state-of-the-art cell type-free predictions and cell type-specific chromatin states to better prioritize regulatory variants. In the composite model, we computed the probability of a causal regulatory variant by the composite likelihood:

$$ P\left( causal\Big| S\right)\kern0.5em =\kern0.5em {\displaystyle \prod_{i=1}^n}\frac{P\left({s}_i\Big| causal\right) \times \pi}{P\left({s}_i\Big| causal\right)\kern0.5em  \times \pi + P\left({s}_i\Big| neutral\right) \times \kern0.5em \left(1\kern0.5em -\kern0.5em \pi \right)\kern0.5em } $$

where *S* is the observed set of prediction scores and the probability that a causal or a neutral SNP obtains a score *s*_*i*_ could be calculated through the empirical distribution of training dataset. The flat prior probability π = 0.5 for the causal probability of each variant.

Given a set of functional prediction scores (*S*) and the chromatin features of one defined cell (*X*) for an observed SNP, one can estimate the posterior probability of the SNP being a regulatory variant. We termed this joint likelihood the “combined probability” as:

$$ P\left( causal\Big| S,\  X\right)\kern0.5em =\kern0.5em \frac{P\left( S,\  X\Big| causal\right) \times \pi}{P\left( S,\  X\right)} $$

Since test scores and chromatin signatures are two independent measurements:

$$ \begin{array}{l} P\left( causal\Big| S,\  X\right) = \kern0.5em \frac{P\left( S\Big| causal\right) P\left( X\Big| causal\right) \times \pi}{P\left( S,\  X\right)}\ \\ {} = \frac{P(S) P(X)\ }{P\left( S,\  X\right)} \times \frac{P\left( causal\Big| S\right) P\left( causal\Big| X\right)\ }{\pi}\ \\ {} = a \times \frac{P\left( causal\Big| S\right) P\left( causal\Big| X\right)\ }{\pi}\end{array} $$

where $a$ is the normalization constant, $P\left( causal\Big| S\right)$ is the likelihood in the composite model and $P\left( causal\Big| X\right)$ is the regulatory potential in the context-dependent model.

### Comparison with other cell type-specific methods

Geuvadis eQTLs of lymphoblastoid cells for 373 European individuals were downloaded from the EMBL-EBI Geuvadis Data Browser (http://www.ebi.ac.uk/Tools/geuvadis-das/). The *cis*-eQTLs from a meta-analysis study of whole blood samples in ~5300 individuals were downloaded from the Blood eQTL Browser (http://www.genenetwork.nl/bloodeqtlbrowser/) [61]. A dataset of RA *cis*-eQTLs with FDR < 5% was retrieved from a published paper [39]. For each of the eQTL datasets, we first removed eQTLs that overlapped with the training eQTL dataset. Next, we used our previous algorithm to compute the composite probability and then used our new combined model to estimate the regulatory probability for all 1000 Genomes variants based on the epigenome annotations of the ENCODE GM12878 lymphoblastoid cell line. For a comparison, we extracted fitCons GM12878 cell type-specific scores for all 1000 Genomes variants. In addition, we computed the deltaSVM GM12878 DHS-weighted scores for all 1000 Genomes variants. Using ranked scores for the four algorithms (composite model, combined model, fitCons, and deltaSVM), we examined how many eQTLs were covered when increasing the percentage of top-ranked variants.

### RA GWAS and eQTL dataset

The RA SNP summary statistics from two GWASs by Okada et al. [44] and by Stahl et al. [46] were retrieved from ImmunoBase [62]. The GWAS by Okada et al. had a large sample size of both European and Asian ancestries consisting of 29,880 cases and 73,758 controls. The GWAS by Stahl et al. [46] had a small sample size consisting of 12,307 cases and 28,975 controls. Another RA eQTL dataset was obtained from a study by Walsh et al. [39], which combined whole-genome sequences and blood transcription profiles of 377 RA patients with over 6000 identified eGenes.

### GWAS signal enrichment

The GWAS signal enrichment analysis was performed using RA-associated SNPs from Okada et al. [44]. SNPs in MHC regions were removed before the analysis. The RA-associated SNPs were prioritized according to the context-dependent combined probability using epigenomic profiles of 13 ENCODE tissues/cell types, including DND41 (Blood), GM12878 (Blood), HeLa-S3 (Cervix), HepG2 (Liver), HMEC (Breast), HSMM (Muscle), HUVEC (Vascular), K562 (Blood), Mo-CD14+ (Blood), NH-A (Brain), NHDF-Ad (Skin), NHLF (Lung), and OSTEO (Bone). For the top 5% prioritized SNPs in each context, we evaluated the GWAS signal enrichment by permutation test. We performed two types of permutation. First, we randomly drew 1 million samples with the same number of top-ranked SNPs from the original GWAS SNPs. To investigate the impact of allele frequency on our results, we further randomly drew one million allele frequency-matched samples with a frequency deviation of less than 0.05 to each top-ranked SNP. We used Fisher’s method to combine GWAS *P* values in each sampling and compared the combined statistics among all samples. To reduce the computational burden, we removed SNPs with GWAS *P* values > 0.001. The Q-Q plot of the top 5% SNPs was constructed to compare the two non-cancer blood cell lines (Mo-CD14+ and GM12878) with other cell lines against the permutated GWAS signals.

### Removal of known RA-associated SNPs

Known RA-associated SNPs were defined according to SNPs reported in GWAS Catalog [4] and SNPs with *P* values < 5 × 10^–8^ from the meta-analysis dataset [44]. These SNPs were removed from the original GWAS dataset, composite dataset, and each context-dependent dataset. The Q-Q plot of the top 5% SNPs was constructed to compare against the permutated GWAS signals as previously mentioned.

### Estimation of effective number of independent markers

We estimated the effective number of independent markers in each SNP set using The Genetic Type I error calculator (GEC) [45], which is a robust tool to remove non-independent SNPs based on LD. For each context, we first compiled two SNP sets: (1) the top 1% SNPs after context-dependent prioritization; and (2) SNPs with sub-genome-wide GWAS significance (*P* values < 1 × 10^–5^) in the top 1% SNPs. For each set, we calculated the effective number (*M*_*e*_) of independent markers chromosome by chromosome. The ratio for each chromosome was obtained as:

$$ Ratio(chr)\kern0.5em =\kern0.5em \frac{M_e\left( sub\hbox{--} genome\hbox{--} wide\  SNPs\right)}{M_e\left( Top\ 1\%\  SNPs\right)} $$

### Weighed GATES gene-based association test

For each RA GWAS SNP from Okada et al. and Stahl et al., we first calculated the context-dependent combined probability using epigenomes of the 13 ENCODE cell lines. The combined probability of SNPs was rescaled to keep the ratio of highest value to the lowest one as ten for each cell line, so that a “wrong” weight would not substantially affect the power [34]. The rescaled probabilities were then used as prior weights of *n* SNPs, ${r}_1,\dots, {r}_n$. According to the procedure of weighted GATES, the final weight of *i*_th_ sorted SNP by its *P* value is

$$ {w}_{(i)}\kern0.5em =\kern0.5em  c\left({m_e}_{(i)}\kern0.5em -\kern0.5em {m_e}_{\left( i-1\right)}\right){r}_{(i)}, $$

where *m*_*e*(*j*)_ is the effective number of independent *P* values among the top *j* SNPs, *m*_*e*(0)_ = 0, and *c* is defined such that the weights sum to *m*_*e*_:

$$ c\kern0.5em =\kern0.5em \frac{m_e}{{\displaystyle {\sum}_{i=0}^m}\left({m}_{e(i)}\kern0.5em -\kern0.5em {m}_{e\left( i-1\right)}\right){r}_{(i)}} $$

The weighted gene-based *P* value was then given by:

$$ {P}_G\kern0.5em =\kern0.5em \mathrm{M}\mathrm{i}\mathrm{n}\left(\frac{m_e{p}_{(j)}}{{\displaystyle {\sum}_{k=1}^j}{w}_{(k)}}\right) $$

For each cell type, we performed the gene-based association test on both the weighted SNPs (W_SNP) and non-weighted SNPs (NW_SNP), respectively. Next, we compared the W_SNP with NW_SNP for each context. For each gene, we defined an improved gene as: (1) a gene where the *P* value decreased from a non-significant level using NW_SNP to a significant level using W_SNP (significant threshold of 1 × 10^–5^); or (2) an already detected gene with a *P* value above a significant level using NW_SNP was further decreased by over 0.8-fold when using W_SNP. A gene detected as significant using NW_SNP but becoming less significant when using W_SNP was defined as a worsened gene.

### Gene set enrichment analysis

Genes from each context-specific prioritization were inputted into the GSEA analysis tool (http://software.broadinstitute.org/gsea) to evaluate the pathway enrichment. The immune system-related gene sets were retrieved from Reactome [63].

## Additional files

Additional file 1: Supplemental Figures and Tables. Supplemental **Figures S1**–**S14** and **Tables S1**–**S14**. (PDF 3992 kb)
Additional file 2: Descriptions and abbreviations of the 127 tissues/cell types. (XLSX 14 kb)
Additional file 3: List of improved genes after context-dependent epigenomic weighting. (XLSX 20 kb)

## Abbreviations

AIC: Akaike information criterion
DHS: DNase I hypersensitive site
eGene: eQTL-associated gene
eQTL: Expression quantitative trait locus
FDR: False discovery rate
GWAS: Genome-wide association study
LD: Linkage disequilibrium
RA: Rheumatoid arthritis
SNP: Single-nucleotide polymorphism
TSS: Transcription start site
