# Supplementary material for: cepip: context-dependent epigenomic weighting for prioritization of regulatory variants and disease-associated genes
Source: Genome Biol. 2017 Mar 16;18:52. doi: 10.1186/s13059-017-1177-3 (PMC5356314; doi:10.1186/s13059-017-1177-3)
Supplement: Additional file 1: — Supplemental Figures and Tables. Supplemental Figures S1–S14 and Tables S1–S14. (PDF 3992 kb) [file 13059_2017_1177_MOESM1_ESM.pdf]

Supplementary figure legends

Fig. S1: Tissue/cell type-specific or generalized logit models trained by selected chromatin features under different controls. Occurrence indicates the number of models sharing the feature during after feature selection procedure; * indicates the *P*-value of coefficient for corresponding feature < 0.05; heatmap color is rendered by exponential coefficients. (A) Strict control; (B) Random control without DHS-related features; and (C) Strict control without DHS-related features.

Fig. S2: Receiver operating characteristic in the low false positive rate spectrum for different generalized context-dependent models. M1: random control (AUC = 0.626); M2: strict control (AUC = 0.619); M3: random control without DHS-related features (AUC = 0.624); M4: strict control without DHS-related features (AUC = 0.609).

Fig. S3: The whole genome XYplot of Log(composite probability) and Log(regulatory potential) using ENCODE GM12878 epigenomic annotations. Pearson correlation coefficient between composite probability and regulatory potential of GM12878 cell line at the genome-wide level shows low dependence (r = 0.197, *P*-value = 2.2×10^{-16}).

Fig. S4: LD proxies of rs12740374 for CEU and YRI populations using 1000 Genomes Phase 1 data.

Fig. S5: The long-range interaction patterns for linked SNPs with the index SNP rs12740374. This figure was plotted using 3DSNP (http://biotech.bmi.ac.cn/3dsnp).

Fig. S6: The Q-Q plots of context-dependent rheumatoid arthritis GWAS signal enrichment for other cell lines. The top 5% SNPs using blood-derived cell (GM12878 and Mo-CD14+)-dependent prioritization shifts leftmost against permutated GWAS signals. Abbreviations: BLD - Blood; LNG - Lung; SKIN - Skin; BONE - Bone; LIV - Liver; VAS - Vascular; MUS - Muscle; BRN - Brain; CRVX - Cervix; BRST - Breast.

Fig. S7: Impact of allele frequency on the results of random sampling. (A) Allele frequency distribution of the context-dependent top 5% SNPs are similar to that of the randomly sampled SNPs without considering allele frequency match. (B) The empirical *P*-values of allele frequency-matched permutations for blood cell lines (Mo-CD14+ and GM12878, orange bars) are more significant than other tissue/cell types. (C) Q-Q plot of the blood (Mo-CD14+ and GM12878), liver (HepG2) and skin (NHDF-Ad) cell lines using allele frequency-matched random sampling.

Fig. S8: Inflation factors (λ) of the top 5% and top 1% SNPs using different cell types. SNPs in the MHC region were removed. Abbreviations: BLD - Blood; LNG - Lung; SKIN - Skin; BONE - Bone; LIV - Liver; VAS - Vascular; MUS - Muscle; BRN - Brain; CRVX - Cervix; BRST – Breast.

Fig. S9: Top-ranked (5%) SNPs display more leftward shift than lower-ranked ones (5-10% or last 5%). (A) Q-Q plot of the blood cell lines (GM12878). (B) Q-Q plot of bone cell lines (OSTEO).

Fig. S10: Similar patterns remain after removing known rheumatoid arthritis loci. (A) Q-Q plots of context-dependent RA GWAS signal enrichment for blood cell line (GM12878) and other non-relevant cell lines. (B) Q-Q plots of context-dependent RA GWAS signal enrichment for blood cell line (Mo-CD14+) and other non-relevant cell lines. (C) The top 5% SNPs display leftmost shifts from permutated GWAS signals compared to lower-ranked ones (5-10% or last 5%) for Mo-CD14+ cells (blood), GM12878 cells (blood) and osteoblast primary cells (bone). Abbreviations: BLD - Blood; LIV - Liver; SKIN - Skin; MUS - Muscle; BONE - Bone.

Fig. S11: Chromatin features around the moderately associated RA SNP rs874628 (GWAS *P*-value = 0.00036). rs874628 was prioritized using blood cell (Mo-CD14+) epigenomic profiles. It is located in the genomic regions enriched with

H3K4me1 and DHS signals (highlighted region), which are specific in blood Mo-CD14+ cells.

Fig. S12: Context-dependent prioritization increases the ratio of effective number of independent markers on all chromosomes. Both context-dependent and composite prioritization significantly increased the ratio compared with original GWAS signals, indicating the context-dependent model improved the discovery of GWAS SNPs by identifying more LD-independent signals. $^{\#}P < 0.05$ compared with original GWAS signal.

Fig. S13: Percentage of improved and worsened genes and eGenes after context-dependent weighting. Using blood cell line (Mo-CD14+ and GM12878)-dependent weighting to detect RA-associated genes, both genes and eGenes showed higher percentages in improved groups and lower percentages in worsened groups.

Fig. S14: Context-dependent epigenomic weighting increases the statistical power of the detection of disease-associated genes. Genes detected using blood cell line-specific W_SNP in the smaller RA cohort tended to be more significant genes than those detected in the larger cohort. The y-axis is the average -log10(*P*-value) of genes detected in the larger cohort. The four blood cell lines are marked in yellow and other cell lines are marked in green.

Supplementary Figures

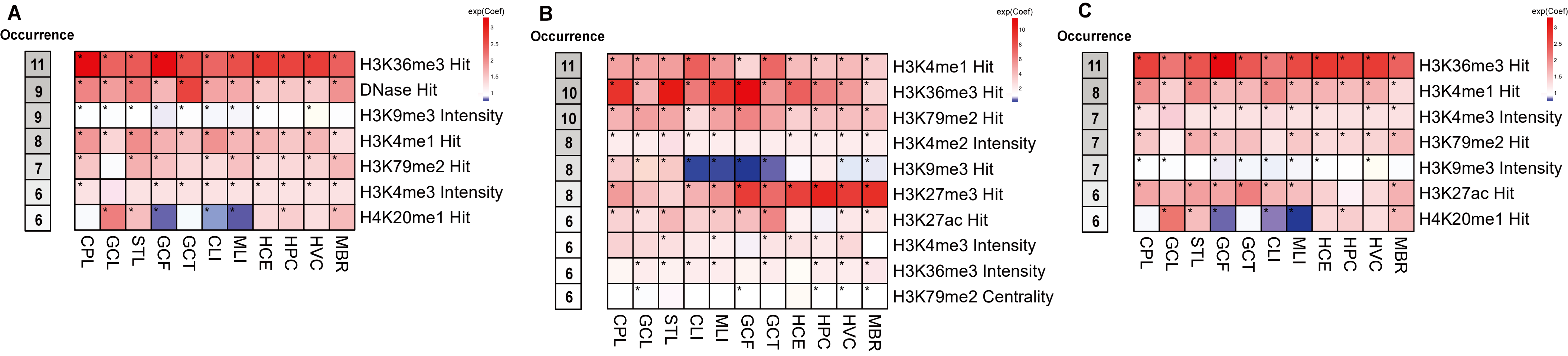

Fig. S1

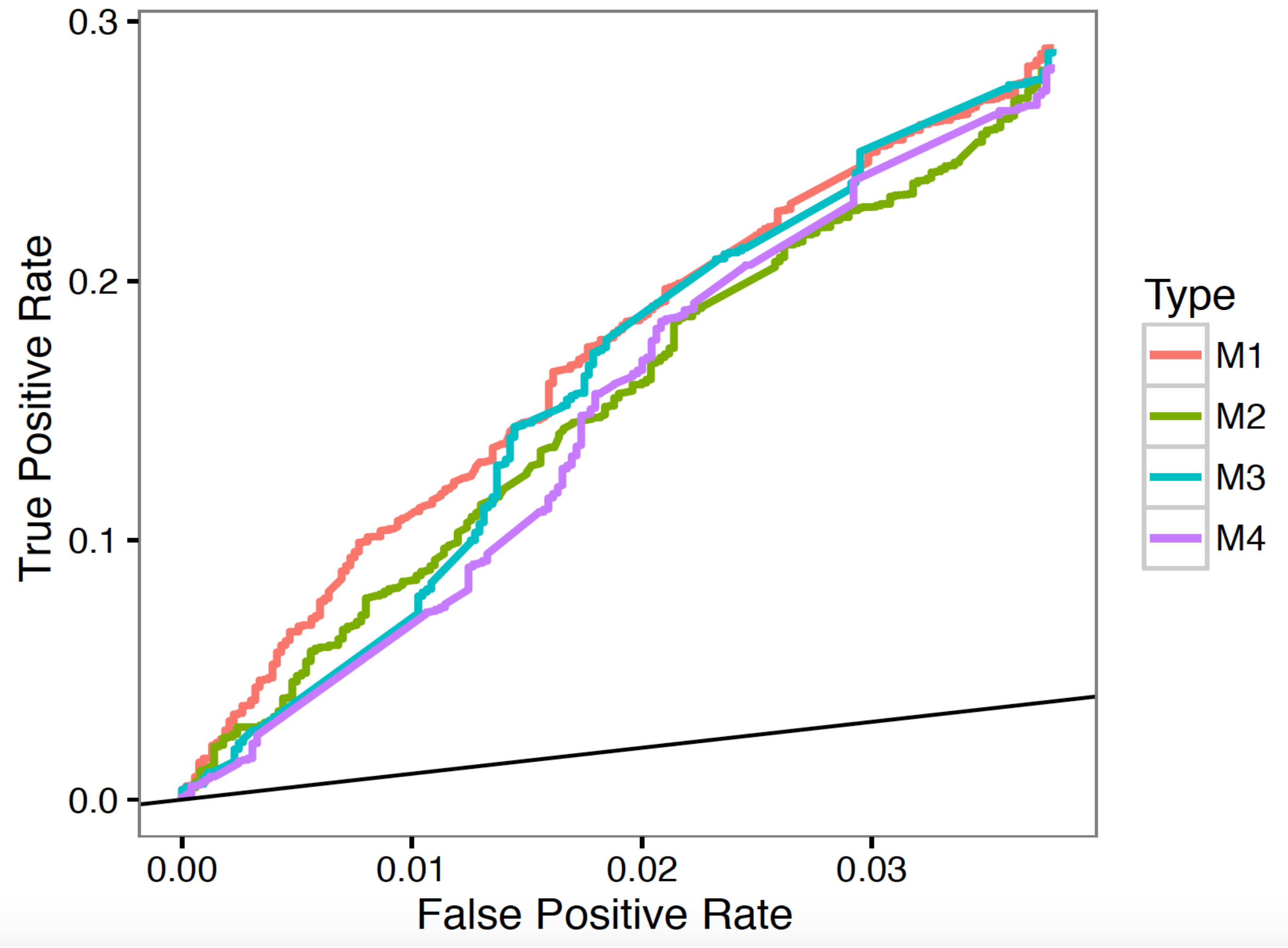
0.3
0.2
0.1
0.0
True Positive Rate
0.00
0.01
0.02
0.03
False Positive Rate
Type
M1
M2
M3
M4

Fig. S2

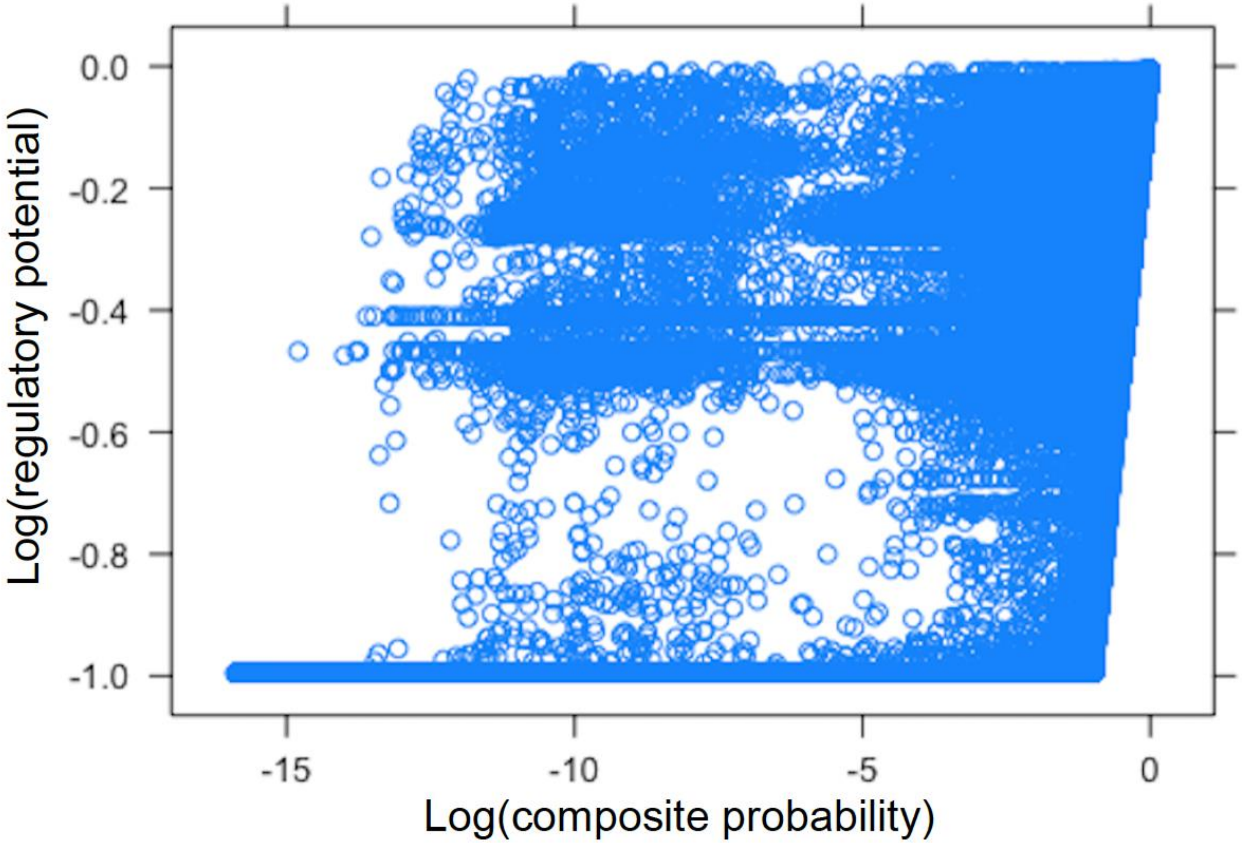
0.0
-0.2
-0.4
-0.6
-0.8
-1.0
Log(regulatory potential)
-15
-10
-5
0
Log(composite probability)

Fig. S3

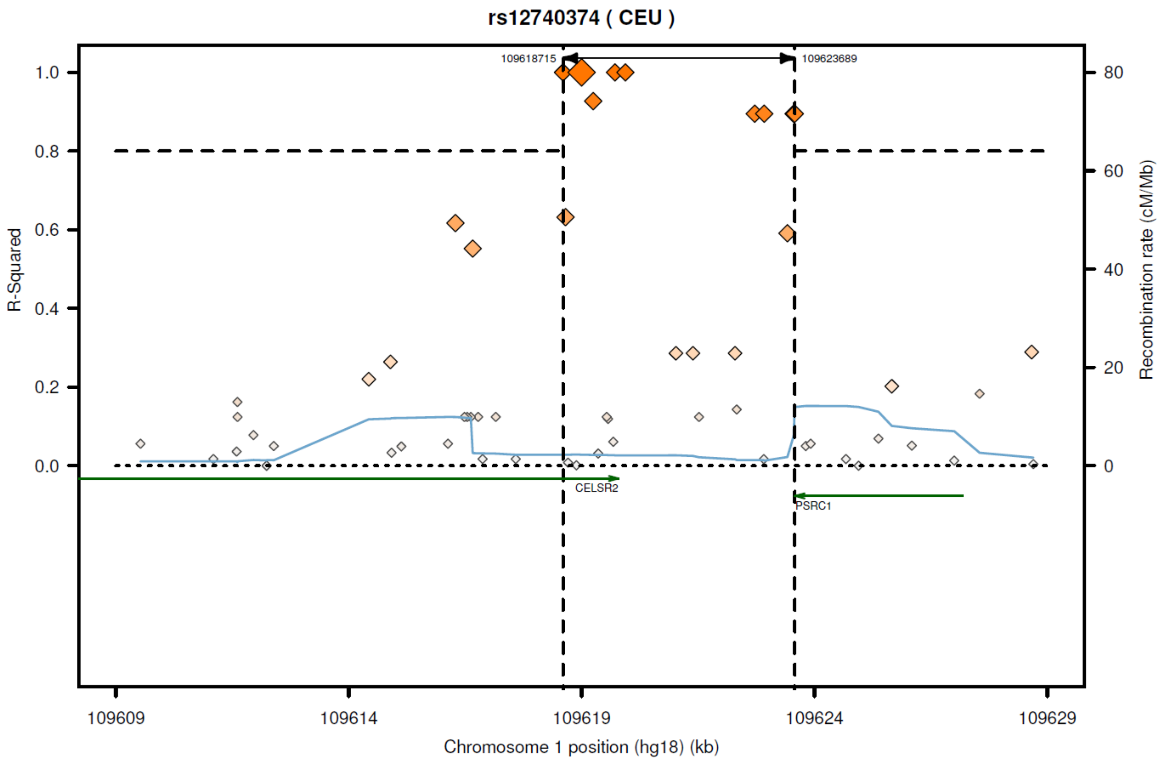
rs12740374 (CEU)
109618715
109623689
R-Squared
Recombination rate (cM/Mb)
CELSR2
PSRC1
Chromosome 1 position (hg18) (kb)

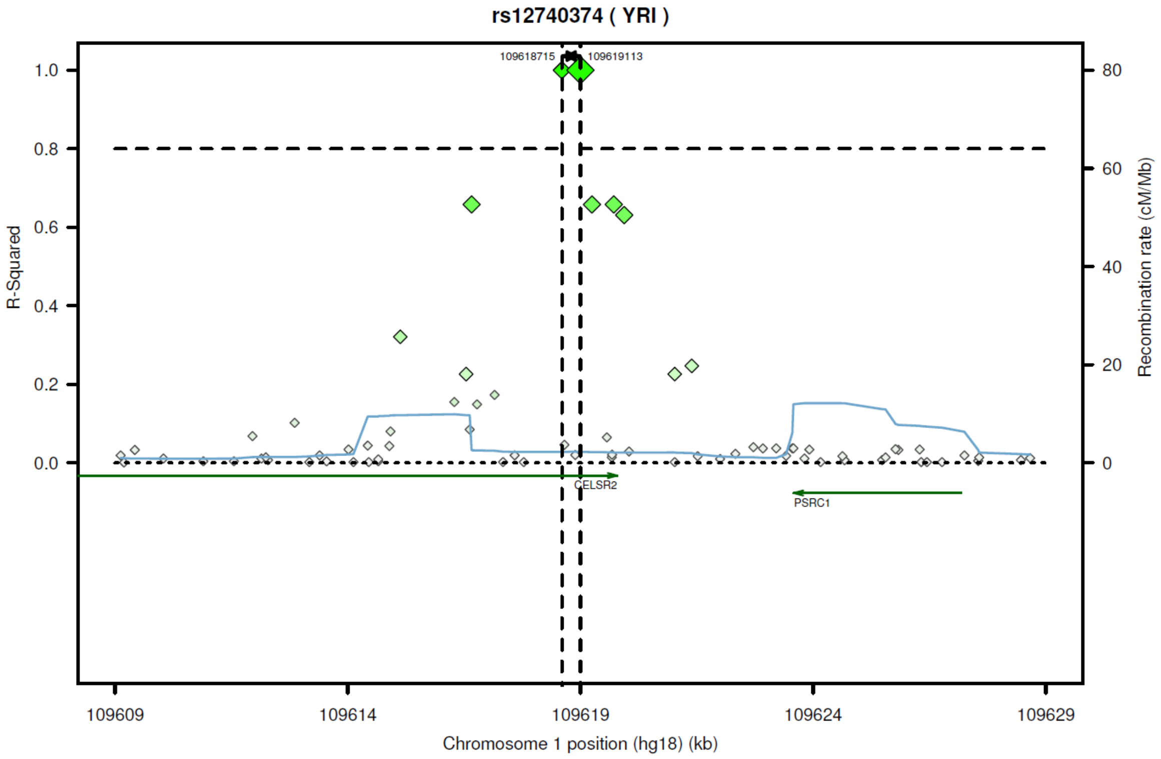
rs12740374 (YRI)
109618715
109619113
R-Squared
Recombination rate (cM/Mb)
CELSR2
PSRC1
Chromosome 1 position (hg18) (kb)

Fig. S4

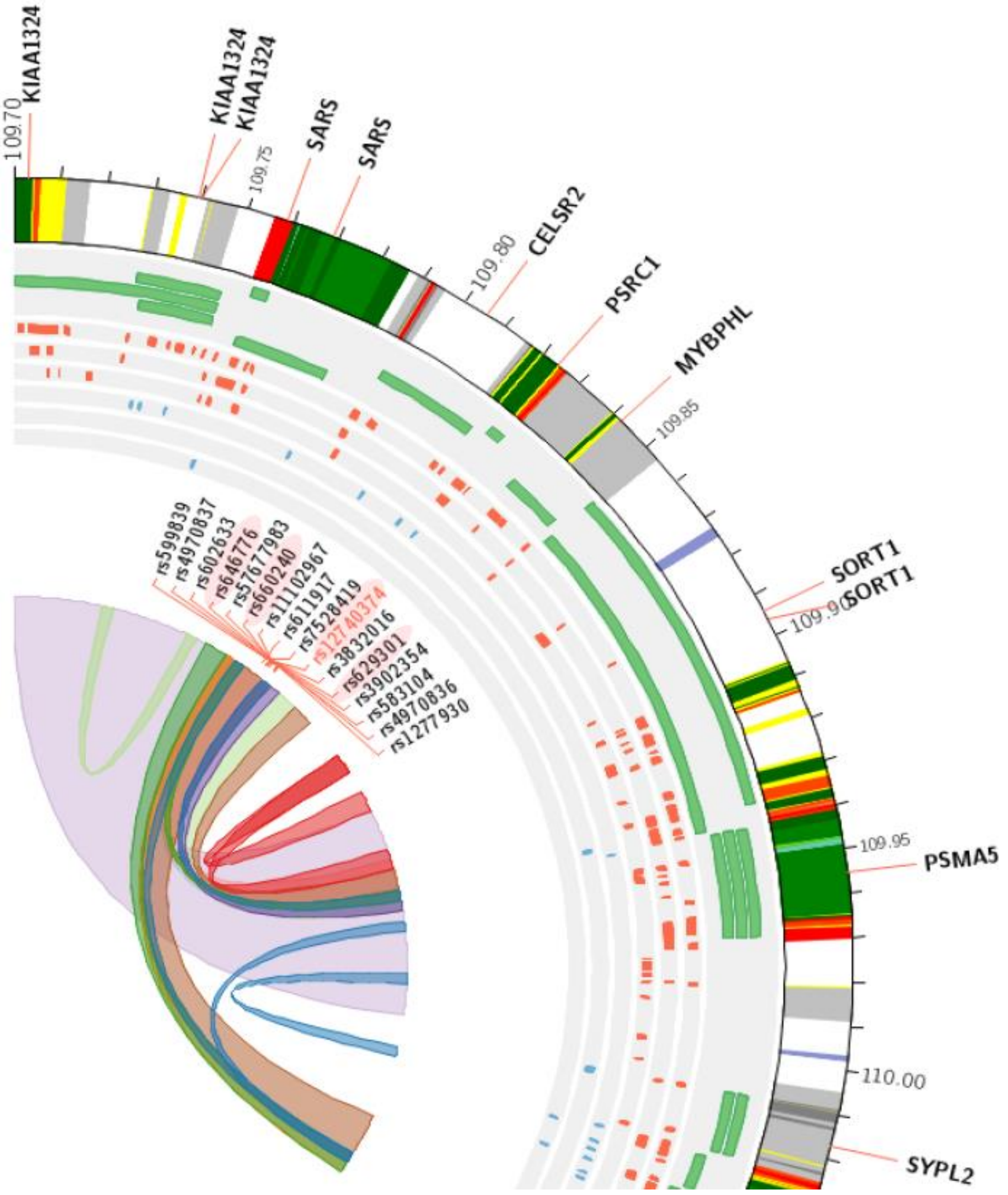

109.70
KIAA1324
KIAA1324
KIAA1324
109.75
SARS
SARS
109.80
CELSR2
PSRC1
MYBPHL
109.85
SORT1
SORT1
109.90
109.95
PSMA5
110.00
SYPL2
rs599839
rs4970837
rs602633
rs646776
rs57677983
rs660240
rs11102967
rs611917
rs7528419
rs12740374
rs3832016
rs629301
rs3902354
rs583104
rs4970836
rs1277930

Fig. S5

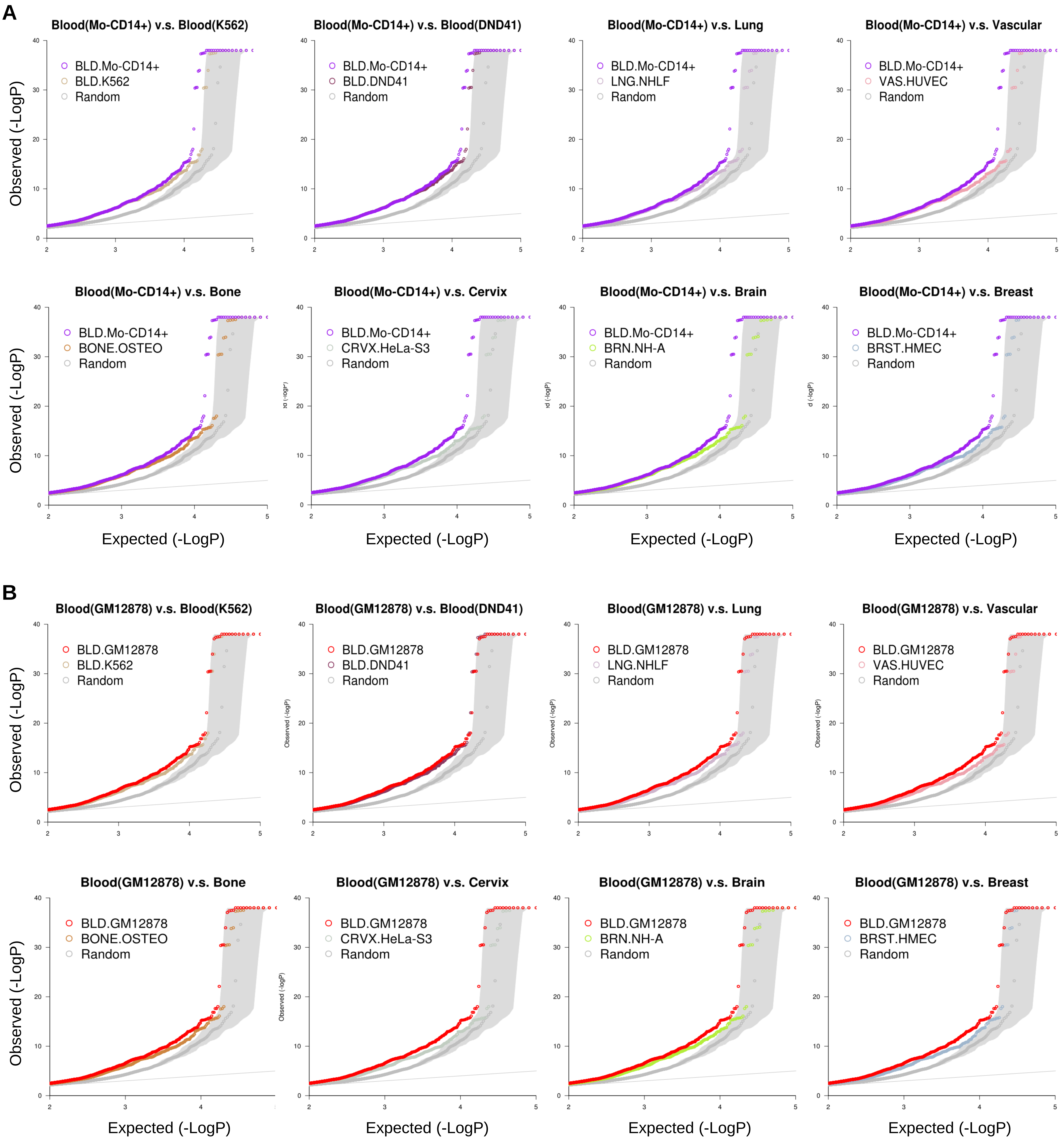
A
Blood(Mo-CD14+) v.s. Blood(K562)
BLD.Mo-CD14+
BLD.K562
Random
Blood(Mo-CD14+) v.s. Blood(DND41)
BLD.Mo-CD14+
BLD.DND41
Random
Blood(Mo-CD14+) v.s. Lung
BLD.Mo-CD14+
LNG.NHLF
Random
Blood(Mo-CD14+) v.s. Vascular
BLD.Mo-CD14+
VAS.HUVEC
Random
Blood(Mo-CD14+) v.s. Bone
BLD.Mo-CD14+
BONE.OSTEO
Random
Blood(Mo-CD14+) v.s. Cervix
BLD.Mo-CD14+
CRVX.HeLa-S3
Random
Blood(Mo-CD14+) v.s. Brain
BLD.Mo-CD14+
BRN.NH-A
Random
Blood(Mo-CD14+) v.s. Breast
BLD.Mo-CD14+
BRST.HMEC
Random
Observed (-LogP)
Expected (-LogP)
B
Blood(GM12878) v.s. Blood(K562)
BLD.GM12878
BLD.K562
Random
Blood(GM12878) v.s. Blood(DND41)
BLD.GM12878
BLD.DND41
Random
Blood(GM12878) v.s. Lung
BLD.GM12878
LNG.NHLF
Random
Blood(GM12878) v.s. Vascular
BLD.GM12878
VAS.HUVEC
Random
Blood(GM12878) v.s. Bone
BLD.GM12878
BONE.OSTEO
Random
Blood(GM12878) v.s. Cervix
BLD.GM12878
CRVX.HeLa-S3
Random
Blood(GM12878) v.s. Brain
BLD.GM12878
BRN.NH-A
Random
Blood(GM12878) v.s. Breast
BLD.GM12878
BRST.HMEC
Random
Observed (-LogP)
Expected (-LogP)

Fig. S6

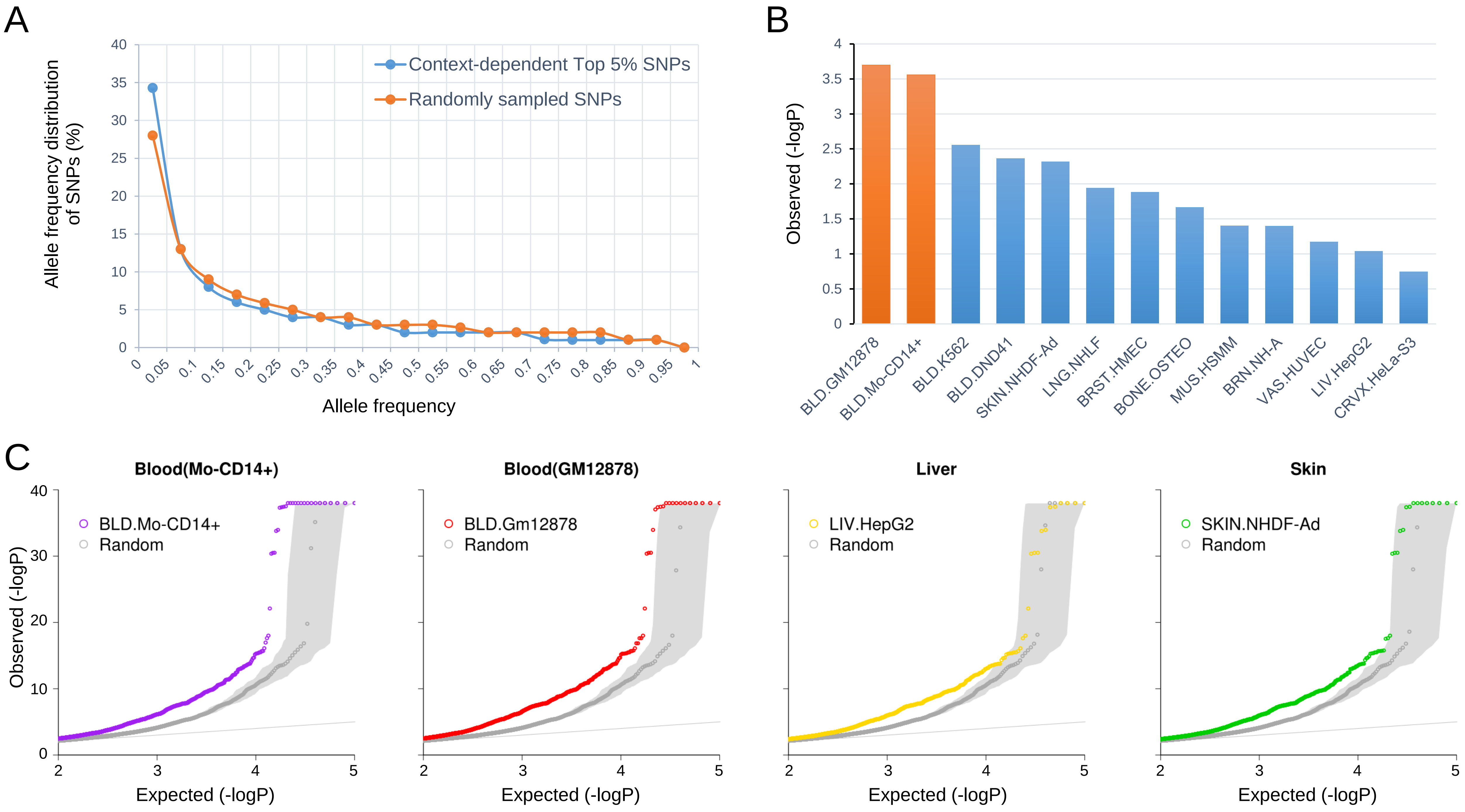

A
Allele frequency distribution of SNPs (%)
Allele frequency
Context-dependent Top 5% SNPs
Randomly sampled SNPs
B
Observed (-logP)
BLD.GM12878
BLD.Mo-CD14+
BLD.K562
BLD.DND41
SKIN.NHDF-Ad
LNG.NHLF
BRST.HMEC
BONE.OSTEO
MUS.HSMM
BRN.NH-A
VAS.HUVEC
LIV.HepG2
CRVX.HeLa-S3
C
Blood(Mo-CD14+)
Blood(GM12878)
Liver
Skin
Observed (-logP)
Expected (-logP)
BLD.Mo-CD14+
BLD.Gm12878
LIV.HepG2
SKIN.NHDF-Ad
Random

Fig. S7

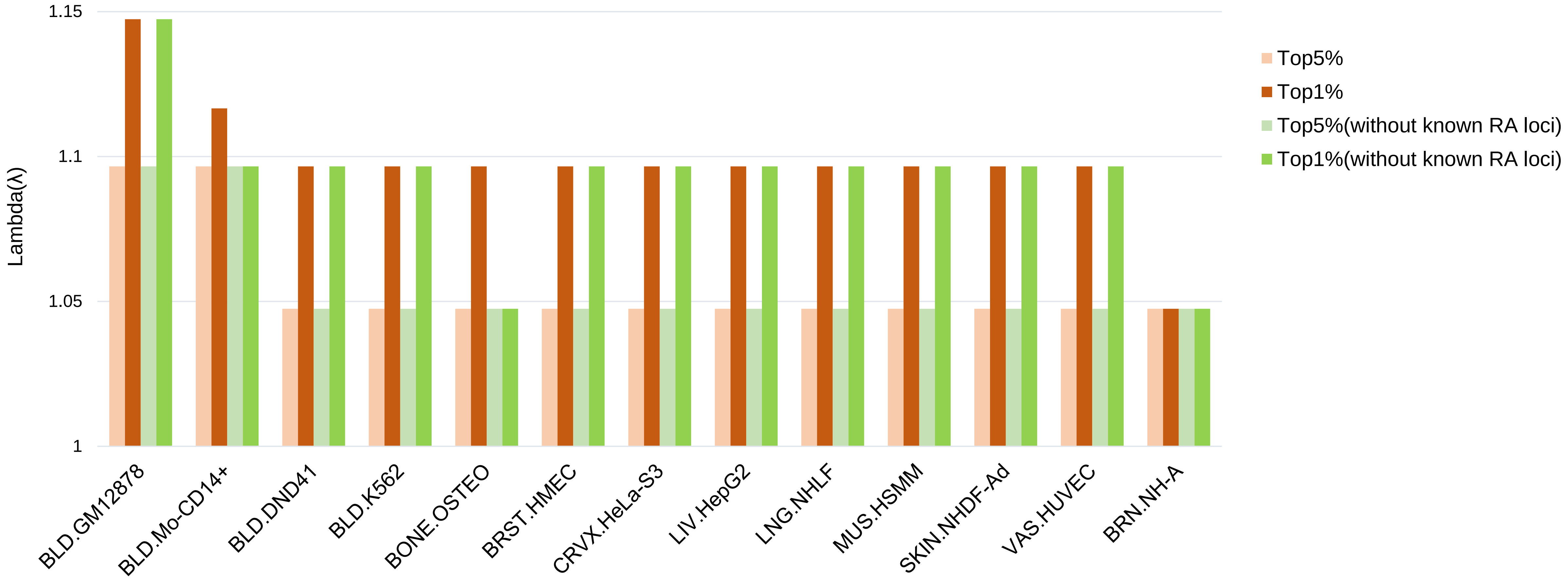

Lambda(λ)
1.15
1.1
1.05
1
BLD.GM12878
BLD.Mo-CD14+
BLD.DND41
BLD.K562
BONE.OSTEO
BRST.HMEC
CRVX.HeLa-S3
LIV.HepG2
LNG.NHLF
MUS.HSMM
SKIN.NHDF-Ad
VAS.HUVEC
BRN.NH-A
Top5%
Top1%
Top5%(without known RA loci)
Top1%(without known RA loci)

Fig. S8

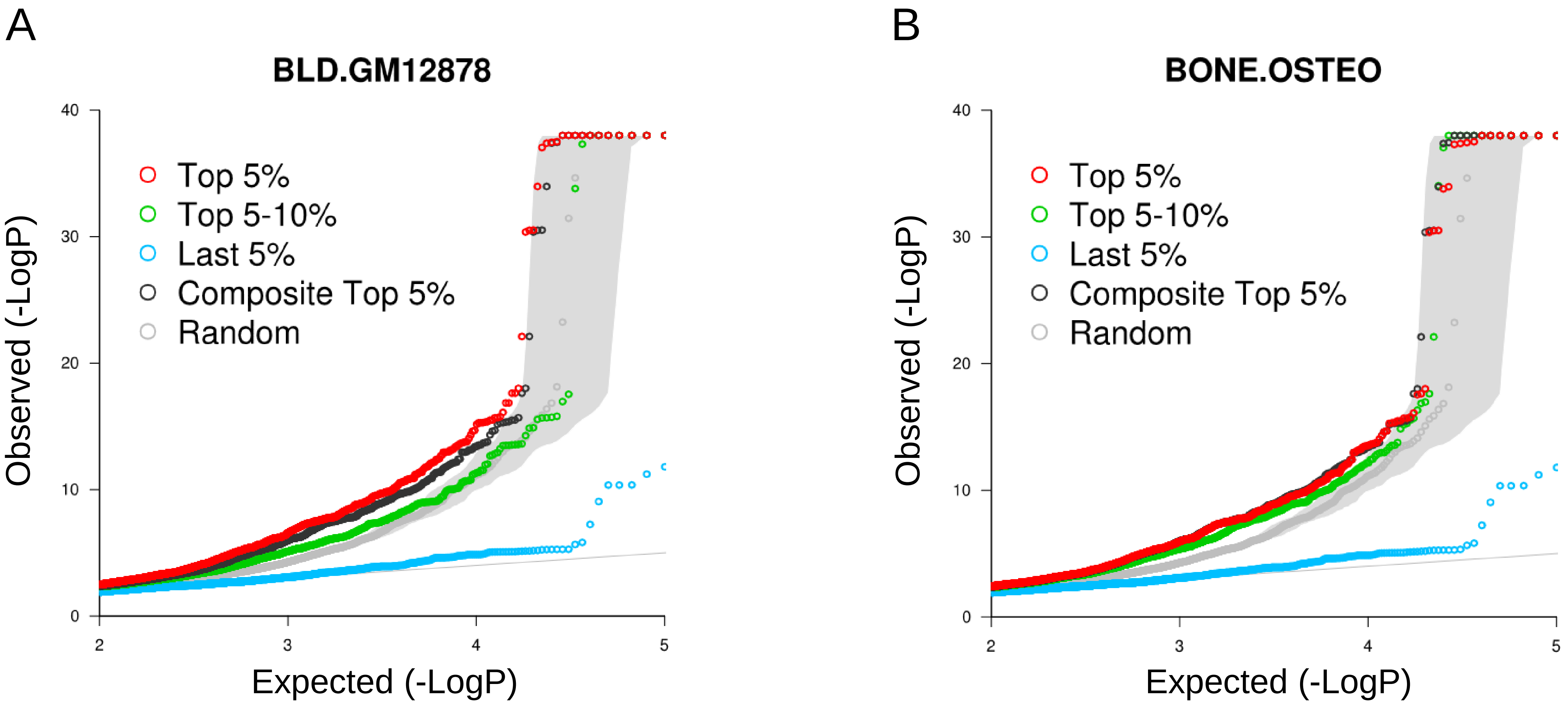
A
BLD.GM12878
Top 5%
Top 5-10%
Last 5%
Composite Top 5%
Random
Observed (-LogP)
Expected (-LogP)
0
10
20
30
40
2
3
4
5
B
BONE.OSTEO
Top 5%
Top 5-10%
Last 5%
Composite Top 5%
Random
Observed (-LogP)
Expected (-LogP)
0
10
20
30
40
2
3
4
5

Fig. S9

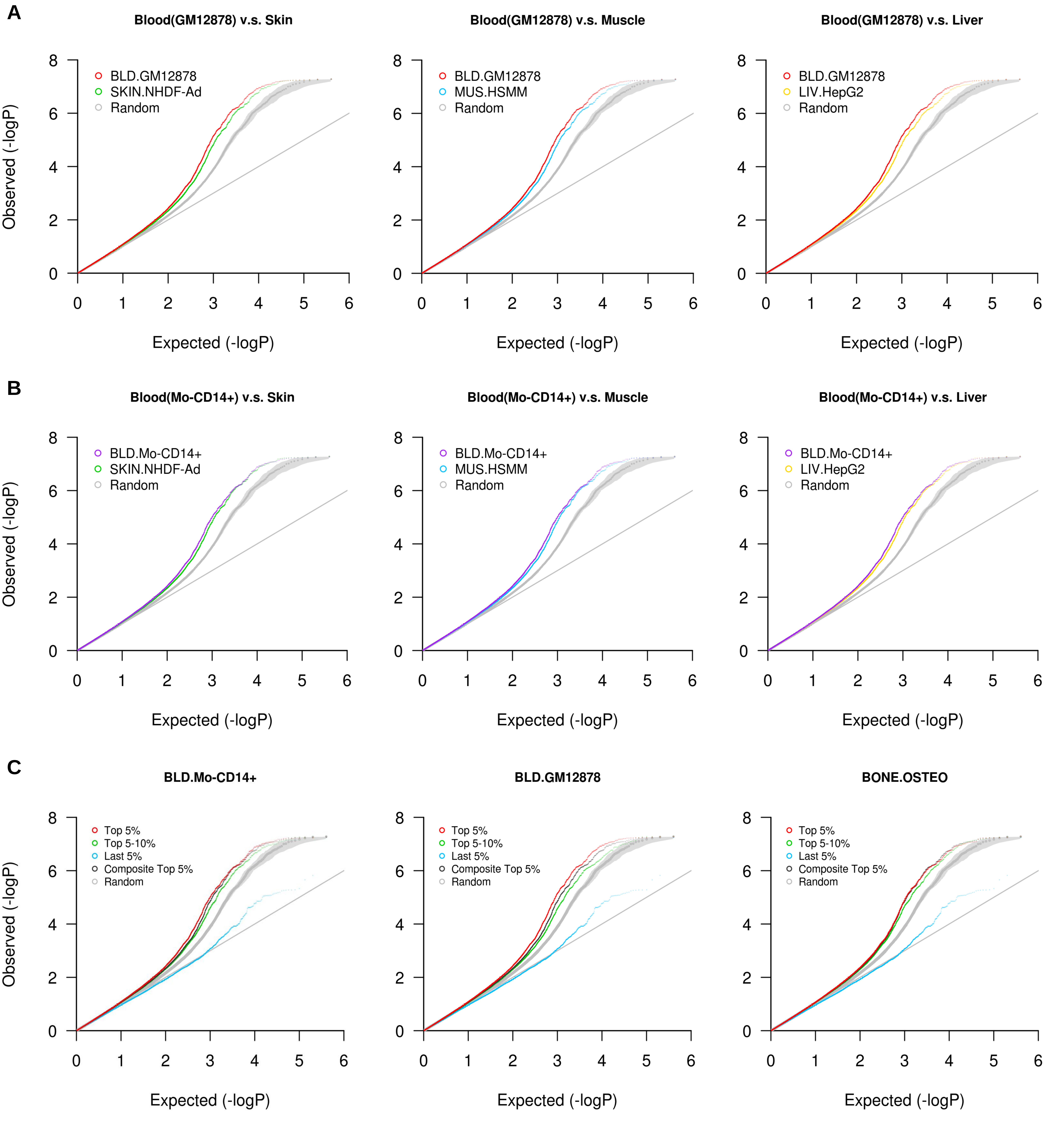
A
Blood(GM12878) v.s. Skin
BLD.GM12878
SKIN.NHDF-Ad
Random
Blood(GM12878) v.s. Muscle
BLD.GM12878
MUS.HSMM
Random
Blood(GM12878) v.s. Liver
BLD.GM12878
LIV.HepG2
Random
B
Blood(Mo-CD14+) v.s. Skin
BLD.Mo-CD14+
SKIN.NHDF-Ad
Random
Blood(Mo-CD14+) v.s. Muscle
BLD.Mo-CD14+
MUS.HSMM
Random
Blood(Mo-CD14+) v.s. Liver
BLD.Mo-CD14+
LIV.HepG2
Random
C
BLD.Mo-CD14+
BLD.GM12878
BONE.OSTEO
Top 5%
Top 5-10%
Last 5%
Composite Top 5%
Random
Observed (-logP)
Expected (-logP)
0
1
2
3
4
5
6
8

Fig. S10

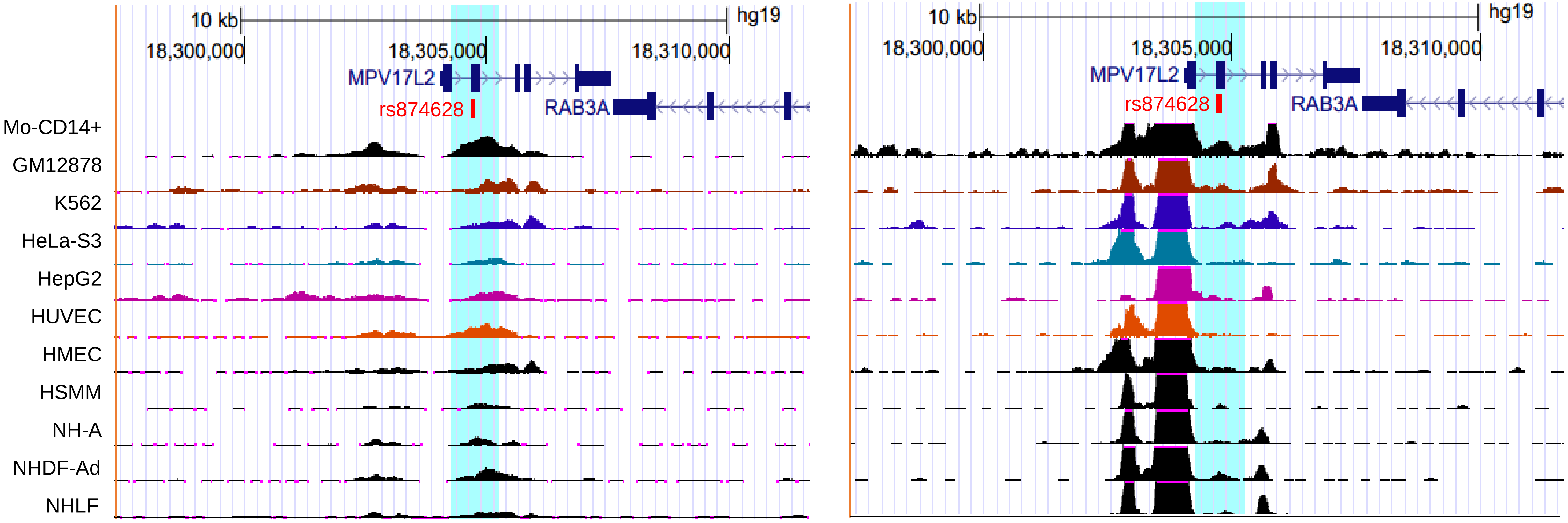

H3K4me1 **DNase I hypersensitivity**

Fig. S11

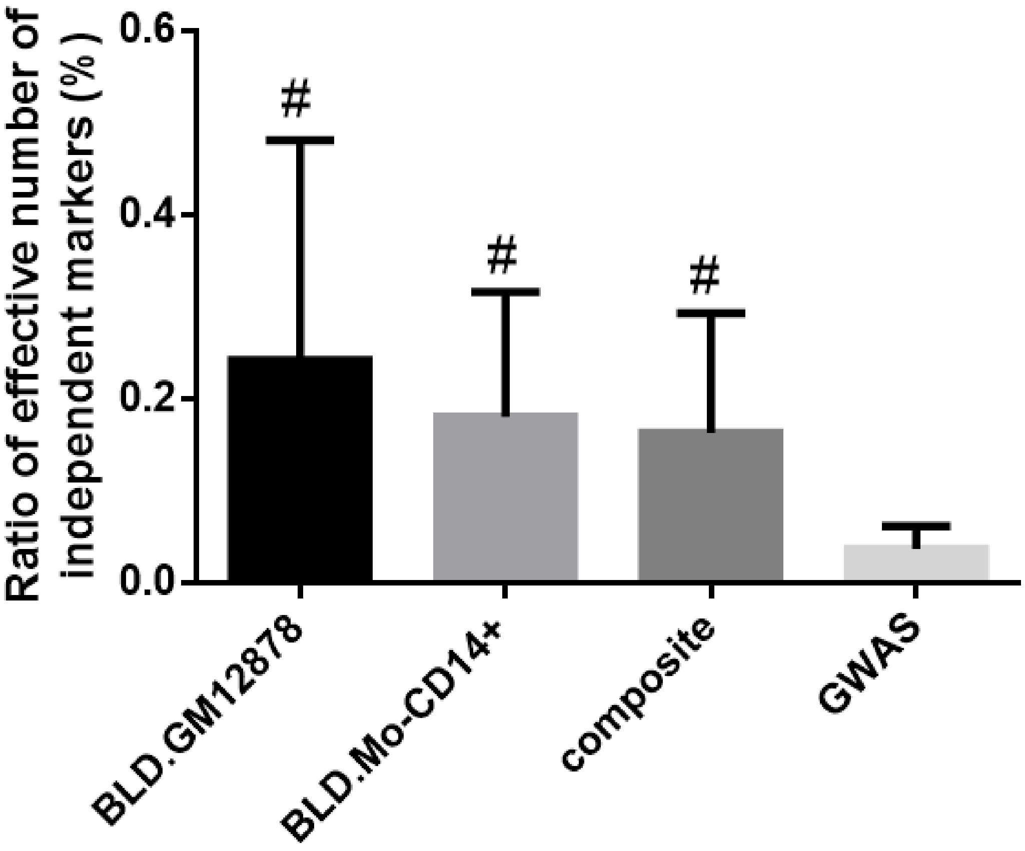
Ratio of effective number of independent markers (%)
0.6
0.4
0.2
0.0
#
#
#
BLD.GM12878
BLD.Mo-CD14+
composite
GWAS

Fig. S12

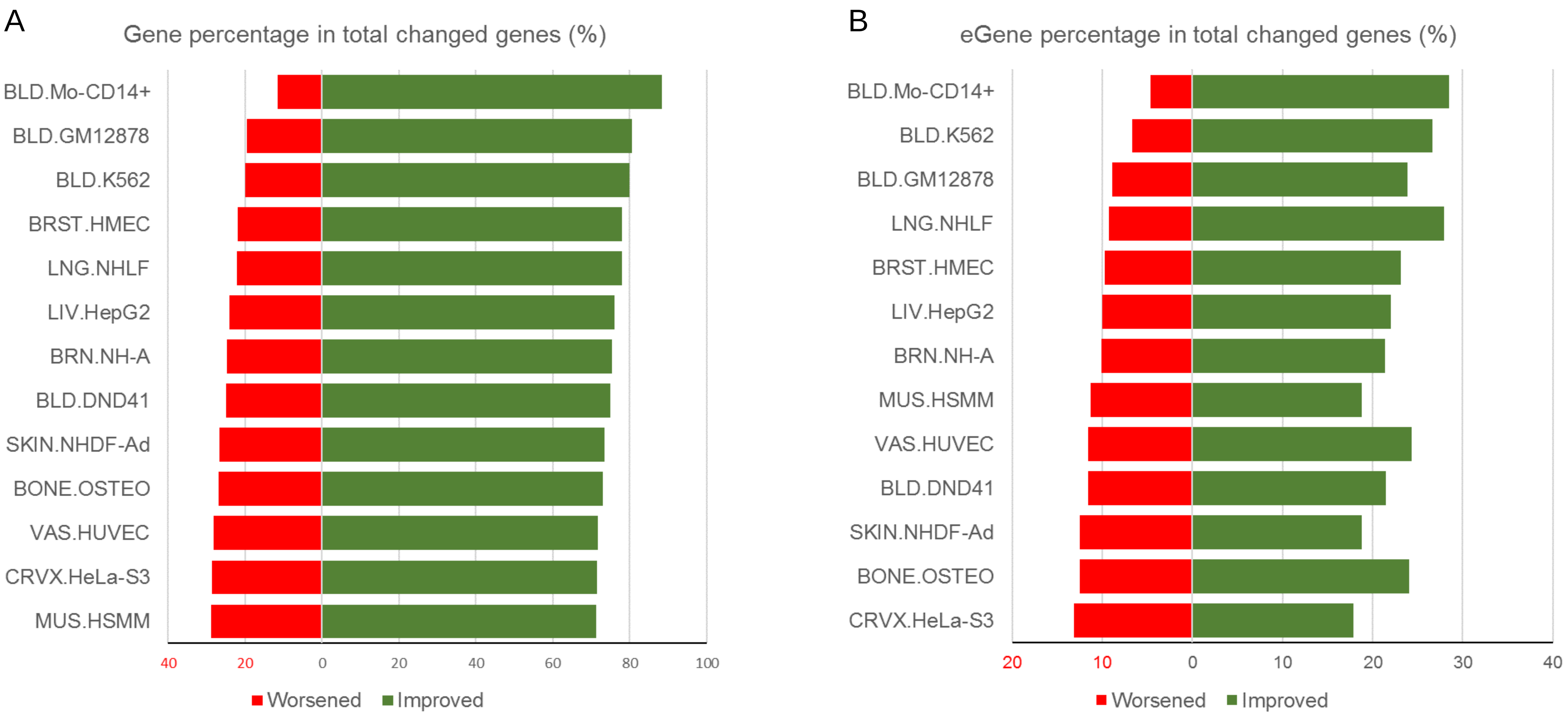

A
Gene percentage in total changed genes (%)
BLD.Mo-CD14+
BLD.GM12878
BLD.K562
BRST.HMEC
LNG.NHLF
LIV.HepG2
BRN.NH-A
BLD.DND41
SKIN.NHDF-Ad
BONE.OSTEO
VAS.HUVEC
CRVX.HeLa-S3
MUS.HSMM
40 20 0 20 40 60 80 100
Worsened
Improved
B
eGene percentage in total changed genes (%)
BLD.Mo-CD14+
BLD.K562
BLD.GM12878
LNG.NHLF
BRST.HMEC
LIV.HepG2
BRN.NH-A
MUS.HSMM
VAS.HUVEC
BLD.DND41
SKIN.NHDF-Ad
BONE.OSTEO
CRVX.HeLa-S3
20 10 0 10 20 30 40
Worsened
Improved

Fig. S13

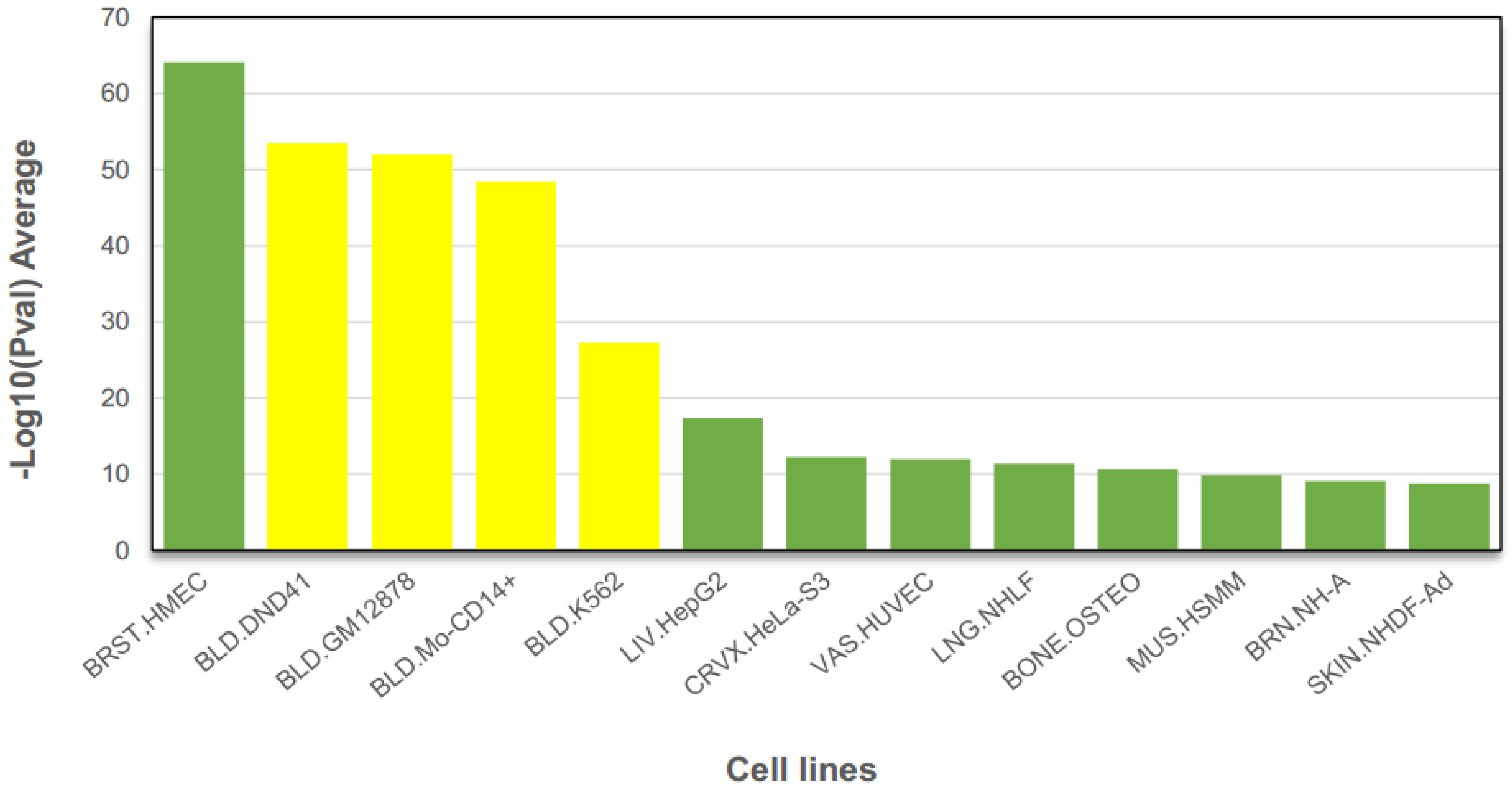

70
60
50
40
30
20
10
0
-Log10(Pval) Average
BRST.HMEC
BLD.DND41
BLD.GM12878
BLD.Mo-CD14+
BLD.K562
LIV.HepG2
CRVX.HeLa-S3
VAS.HUVEC
LNG.NHLF
BONE.OSTEO
MUS.HSMM
BRN.NH-A
SKIN.NHDF-Ad
Cell lines

Fig. S14

Supplementary Tables

Table S1: 11 eQTL datasets and matched ENCODE cell lines.

Abbr.	eQTL Study	Tissues/Cells	Fine Mapped eQTLs (10% FDR)	ENCODE Mapped Cell Line
**CPL**	CAP_LCL	Lymphoblastoid cell line	20,863	GM12878
**GCL**	GenCord_LCL	Lymphoblastoid cell line	2,390	GM12878
**STL**	Stranger_LCL	Lymphoblastoid cell line	12,117	GM12878
**GCF**	GenCord_fibroblast	Blood fibroblast	2,702	HUVEC
**GCT**	GenCord_tcell	Blood T cell	2,258	DND41
**CLI**	UChicago_liver	Liver tissue	9,388	HepG2
**MLI**	Merck_liver	Liver tissue	9,137	HepG2
**HCE**	Harvard_cerebellum	Brain cerebellum	15,086	NH-A
**HPC**	Harvard_prefrontal_cortex	Brain prefrontal cortex	19,209	NH-A
**HVC**	Harvard_visual_cortex	Brain visual cortex	13,450	NH-A
**MBR**	Myers_brain	Brain cortical	3,338	NH-A
**Geuvadis**	Geuvaids_LCL	Lymphoblastoid cell line	6,685	GM12878

Table S2: Occurrence of selected chromatin features in tissue/cell type-specific logit models. This table shows results from 11 eQTL datasets with random control around TSS.

Feature	Occurrence
**H3K4me1 Hit**	**11**
**H3K36me3 Hit**	**11**
**DHS Hit**	**10**
**H3K79me2 Hit**	**10**
**H3K9me3 Hit**	**9**
**H3K27me3 Hit**	**8**
**H3K4me2 Intensity**	**7**
**H3K36me3 Intensity**	**6**
**H3K4me3 Intensity**	**6**
**H3K79me2 Centrality**	**6**
H4K20me1 Hit	5
H3K4me2 Hit	5
H3K9ac Intensity	5
H3K9me3 Intensity	5
H2AZ Centrality	5
H3K27ac Intensity	4
H3K27me3 Intensity	4
H3K4me1 Intensity	4
H3K27me3 Centrality	4
H2AZ Hit	3
H4K20me1 Intensity	3
DHS Centrality	3
H3K4me2 Centrality	3
H3K27ac Hit	2
H3K9ac Hit	2
DHS Intensity	2
H2AZ Intensity	2
H3K27ac Centrality	2
H3K36me3 Centrality	2
H3K4me1 Centrality	2
H3K4me3 Centrality	2
H3K9ac Centrality	2
H3K9me3 Centrality	2
H4K20me1 Centrality	2
H3K4me3 Hit	1
H3K79me2 Intensity	1

Table S3: Occurrence of selected chromatin features in cell type-specific logit models. This table shows results using 11 eQTL datasets with strict control.

Feature	Occurrence
**H3K36me3 Hit**	**11**
**DHS Hit**	**9**
**H3K9me3 Intensity**	**9**
**H3K4me1 Hit**	**8**
**H3K79me2 Hit**	**7**
**H4K20me1 Hit**	**6**
**H3K4me3 Intensity**	**6**
H3K27ac Hit	5
H3K4me2 Intensity	5
H4K20me1 Intensity	5
DHS Centrality	5
H3K4me3 Centrality	5
H3K9me3 Hit	4
DHS Intensity	4
H2AZ Centrality	4
H3K27me3 Centrality	4
H3K4me2 Centrality	4
H3K27me3 Hit	3
H3K4me2 Hit	3
H3K27ac Centrality	3
H3K36me3 Centrality	3
H3K4me1 Centrality	3
H3K9ac Centrality	3
H3K9me3 Centrality	3
H2AZ Hit	2
H3K9ac Hit	2
H2AZ Intensity	2
H3K27ac Intensity	2
H3K27me3 Intensity	2
H3K36me3 Intensity	2
H3K79me2 Intensity	2
H3K9ac Intensity	2
H3K4me1 Intensity	1
H3K79me2 Centrality	1
H4K20me1 Centrality	1

Table S4: Occurrence of selected chromatin features (trained without DHS marks) in cell type-specific logit models. This table shows results using 11 eQTL datasets with random control around TSS.

**Feature**	**Occurrence**
**H3K4me1 Hit**	**11**
**H3K36me3 Hit**	**10**
**H3K79me2 Hit**	**10**
**H3K27me3 Hit**	**8**
**H3K4me2 Intensity**	**8**
**H3K9me3 Hit**	**8**
**H3K27ac Hit**	**6**
**H3K36me3 Intensity**	**6**
**H3K4me3 Intensity**	**6**
**H3K79me2 Centrality**	**6**
H3K4me2 Hit	5
H3K9ac Intensity	5
H3K9me3 Intensity	5
H4K20me1 Hit	5
H2AZ Hit	4
H3K27me3 Centrality	4
H3K27me3 Intensity	4
H3K4me1 Intensity	4
H2AZ Centrality	3
H3K27ac Centrality	3
H3K4me2 Centrality	3
H3K9ac Centrality	3
H3K9me3 Centrality	3
H2AZ Intensity	2
H3K27ac Intensity	2
H3K36me3 Centrality	2
H3K4me1 Centrality	2
H3K4me3 Centrality	2
H3K4me3 Hit	2
H4K20me1 Centrality	2
H4K20me1 Intensity	2
H3K79me2 Intensity	1
H3K9ac Hit	1

Table S5: Occurrence of selected chromatin features (trained without DHS marks) in cell type-specific logit models. This table shows results using 11 eQTL datasets with strict control.

Feature	Occurrence
**H3K36me3 Hit**	**11**
**H3K4me1 Hit**	**8**
**H3K4me3 Intensity**	**7**
**H3K79me2 Hit**	**7**
**H3K9me3 Intensity**	**7**
**H3K27ac Hit**	**6**
**H4K20me1 Hit**	**6**
H4K20me1 Intensity	6
H3K4me2 Hit	5
H3K4me2 Intensity	5
H3K4me3 Centrality	5
H3K9me3 Hit	5
H2AZ Centrality	4
H2AZ Hit	4
H3K27me3 Centrality	4
H3K27me3 Hit	4
H3K4me2 Centrality	4
H3K9ac Centrality	4
H3K27ac Centrality	3
H3K36me3 Centrality	3
H3K9ac Hit	3
H2AZ Intensity	2
H3K27ac Intensity	2
H3K27me3 Intensity	2
H3K36me3 Intensity	2
H3K4me1 Centrality	2
H3K79me2 Centrality	2
H3K79me2 Intensity	2
H3K9ac Intensity	2
H3K9me3 Centrality	2
H3K4me1 Intensity	1

Table S6: 13 GTEx eQTL datasets and matched Roadmap Epigenomics of tissues/cell lines.

Abbr	GTEx Tissues	Tissues/Cells	GTEx single tissue eQTLs	127 Epigenomes Mapped Tissues/Cell Lines
**AS**	Adipose_Subcutaneous	Adipose Nuclei	105,321	E063
**AA**	Artery_Aorta	Aorta	58,142	E065
**AT**	Artery_Tibial	Blood fibroblast	151,755	E122
**EMA**	Esophagus_Mucosa	Esophagus	99,548	E079
**EMS**	Esophagus_Muscularis	Esophagus	109,017	E079
**HLV**	Heart_Left_Ventricle	Left Ventricle	64,750	E095
**LU**	Lung	NHLF Lung Fibroblast Primary Cells	126,603	E128
**MS**	Muscle_Skeletal	HSMM cell derived Skeletal Muscle Myotubes Cell Line	115,021	E121
**NT**	Nerve_Tibial	H9 Derived Neuron Cultured Cells	125,215	E010
**SSELL**	Skin_Sun_Exposed_Lower_leg	NHDF-Ad Adult Dermal Fibroblast Primary Cells	117,171	E126
**ST**	Stomach	Fetal Stomach	40,140	E092
**TH**	Thyroid	Thymus	162,016	E112
**WB**	Whole_Blood	Lymphoblastoid Cell Line	145,917	E116

Table S7: Occurrence of selected chromatin features in cell type-specific logit models. This table shows results using 13 GTEx eQTL datasets.

**Feature**	**Occurrence**
**DHS Hit**	**13**
**H3K4me1 Hit**	**12**
**H3K36me3 Hit**	**11**
**H3K79me2 Hit**	**11**
**H3K27me3 Hit**	**10**
**H3K4me3 Intensity**	**9**
**H3K4me2 Intensity**	**8**
**H3K9me3 Intensity**	**8**
**H3K79me2 Centrality**	**7**
**H3K27AC Intensity**	**7**
**DHS Centrality**	**7**
H3K9me3 Hit	6
H3K36me3 Intensity	6
H4K20me1 Hit	6
H2AZ Centrality	6
H3K9ac Intensity	5
H3K27me3 Intensity	5
H3K4me1 Intensity	5
H3K27me3 Centrality	5
H2AZ Hit	4
H4K20me1 Intensity	4
H3K4me2 Centrality	4
H3K27AC Hit	3
H3K9ac Hit	3
DHS Intensity	3
H3K36me3 Centrality	3
H3K4me2 Hit	3
H3K4me3 Centrality	3
H2AZ Intensity	2
H3K27AC Centrality	2
H3K4me1 Centrality	2
H3K9ac Centrality	2
H3K9me3 Centrality	2
H3K4me3 Hit	2
H4K20me1 Centrality	1
H3K79me2 Intensity	1

Table S8: The model parameters for generalized condition-dependent model.

Parameters	Coefficients	EXP (Coef)	Std. Error	Z value	P value	Significance
**(Intercept)**	-5.34E-01	5.86E-01	8.15E-03	-65.513	< 2E-16	***
**H3K4me1 Hit**	1.05E+00	2.86E+00	4.11E-02	25.554	< 2E-16	***
**H3K36me3 Hit**	1.57E+00	4.79E+00	8.27E-02	18.932	< 2E-16	***
**DHS Hit**	1.21E+00	3.36E+00	4.87E-02	24.937	< 2E-16	***
**H3K79me2 Hit**	9.75E-01	2.65E+00	4.52E-02	21.577	< 2E-16	***
**H3K9me3 Hit**	-4.84E-01	6.16E-01	5.81E-02	-8.334	< 2E-16	***
**H3K27me3 Hit**	1.52E+00	4.55E+00	6.48E-02	23.389	< 2E-16	***
**H3K4me2 Intensity**	8.69E-04	1.00E+00	9.81E-05	8.86	< 2E-16	***
**H3K4me3 Intensity**	3.09E-04	1.00E+00	7.81E-05	3.955	7.66E-05	***
**H3K36me3 Intensity**	4.35E-03	1.00E+00	1.12E-03	3.897	9.73E-05	***
**H3K79me2 Centrality**	-1.50E-04	1.00E+00	3.06E-05	-4.896	9.78E-07	***

Table S9: The top-mapped tissues/cell types among 38 immune and non-immune diseases/traits.

Disease	Disease/Trait	Epigenome Mnemonic	Epigenome Name	Anatomy
Autoimmune Disease	C reactive protein	ESDR.CD184.ENDO	hESC Derived CD184+ Endoderm Cultured Cells	ESC_DERIVED
	Vitiligo	BLD.CD19.PPC	Primary B cells from peripheral blood	BLOOD
	Asthma	BLD.CD4.CD25M.IL17P.PL.TPC	Primary T helper 17 cells PMA-I stimulated	BLOOD
	Atopic dermatitis	BLD.CD4.MPC	Primary T helper memory cells from peripheral blood 2	BLOOD
	Allergy	BLD.CD4.MPC	Primary T helper memory cells from peripheral blood 2	BLOOD
	Rheumatoid arthritis	BLD.GM12878	GM12878 Lymphoblastoid Cell Line	BLOOD
	Juvenile idiopathic arthritis	BLD.CD4.CD25.CD127M.TREGPC	Primary T regulatory cells from peripheral blood	BLOOD
	Type 1 diabetes	BLD.CD3.PPC	Primary T cells from peripheral blood	BLOOD
	Autoimmune thyroiditis	BLD.CD19.CPC	Primary B cells from cord blood	BLOOD
	Behcets disease	BLD.DND41.CNCR	Dnd41 TCell Leukemia Cell Line	BLOOD
	Primary sclerosing cholangitis	ADRL.GLND.FET	Fetal Adrenal Gland	ADRENAL
	Primary biliary cirrhosis	BLD.GM12878	GM12878 Lymphoblastoid Cell Line	BLOOD
	Multiple sclerosis	BLD.CD3.PPC	Primary T cells from peripheral blood	BLOOD
	Celiac disease	BLD.CD3.CPC	Primary T cells from peripheral blood	BLOOD
	Systemic lupus erythematosus	BLD.GM12878	GM12878 Lymphoblastoid Cell Line	BLOOD
	Systemic sclerosis	BLD.CD4.CD25M.IL17P.PL.TPC	Primary T helper 17 cells PMA-I stimulated	BLOOD
	Ankylosing spondylitis	BLD.CD14.MONO	Monocytes-CD14+ RO01746 Cell Line	BLOOD
	Alopecia areata	BLD.CD4.CD25.CD127M.TREGPC	Primary T regulatory cells from peripheral blood	BLOOD
	Psoriasis	BLD.GM12878	GM12878 Lymphoblastoid Cell Line	BLOOD
	Crohns disease	BLD.CD4.CD25M.CD45RA.NPC	Primary T helper naive cells from peripheral blood	BLOOD
	Ulcerative colitis	SKIN.PEN.FRSK.FIB.02	Foreskin Fibroblast Primary Cells skin02	SKIN
	Kawasaki disease	BLD.DND41.CNCR	Dnd41 TCell Leukemia Cell Line	BLOOD
Non-Autoimmune Disease	Renal function related traits	KID.FET	Fetal Kidney	KIDNEY
	Liver enzyme levels gamma glutamyl transferase	GI.L.INT.FET	Fetal Intestine Large	INTESTINE
	Urate levels	LIV.HEPG2.CNCR	HepG2 Hepatocellular Carcinoma Cell Line	LIVER
	Bone mineral density	ESC.I3	ES-I3 Cell Line	ESC
	Type 2 diabetes	BRST.HMEC	HMEC Mammary Epithelial Primary Cells	BREAST
	Alzheimer's combined	BRN.ANG.GYR	Brain Angular Gyrus	BRAIN
	Restless legs syndrome	BRN.FET.M	Fetal Brain Male	BRAIN
	HDL cholesterol	LIV.HEPG2.CNCR	HepG2 Hepatocellular Carcinoma Cell Line	LIVER
	LDL cholesterol	LIV.ADLT	Adult Liver	LIVER
	Triglycerides	LIV.ADLT	Adult Liver	LIVER
	Fasting glucose related traits	FAT.ADIP.DR.MSC	Adipose Derived Mesenchymal Stem Cell Cultured Cells	FAT
	Progressive supranuclear palsy	IPSC.20B	iPS-20b Cell Line	IPSC
	Red blood cell traits	BLD.K562.CNCR	K562 Leukemia Cell Line	BLOOD
	Platelet counts	PLCNT.FET	Placenta	PLACENTA
	Creatinine levels	SKIN.PEN.FRSK.FIB.01	Foreskin Fibroblast Primary Cells skin01	SKIN
	Migraine	SKIN.PEN.FRSK.MEL.03	Foreskin Melanocyte Primary Cells skin03	SKIN

Table S10: The prioritization result for 17 LDL-C-associated SNPs using the ENCODE HepG2 epigenome. Base on combined probability, SNP rs12740374 (bold) was ranked fifth highest in our final prioritization list.

Chr	Pos	SNP_Id	GWAS_P[1]	Cell_P[2]	BF[3]	Composite_P[4]	Combined_P[5]
1	109826136	rs657420	1.30E-09	0.805254	106294.4	0.138369	0.222845
1	109817838	rs660240	8.30E-41	0.888678	146693.7	0.10557	0.187636
1	109818530	rs646776	2.20E-41	0.888678	10592.66	0.060044	0.10672
1	109818306	rs629301	2.20E-41	0.703519	7746.132	0.063475	0.089312
1	109817590	**rs12740374**	1.80E-42	0.73613	10149.74	0.059073	0.086971
1	109815252	rs611917	8.90E-29	0.36963	1695.044	0.060724	0.044891
1	109820919	rs17035949	1.00E-06	0.626575	2727.231	0.03028	0.037945
1	109827253	rs672569	2.00E-14	0.36963	1217.414	0.050151	0.037075
1	109814880	rs4970834	1.60E-25	0.36963	933.5832	0.048641	0.035959
1	109807283	rs6657811	3.30E-22	0.36963	76.95873	0.020686	0.015293
1	109813719	rs17035665	0.00047	0.36963	87.71737	0.015119	0.011177
1	109821511	rs602633	7.60E-41	0.626575	30.79655	0.008176	0.010246
1	109822166	rs599839	7.30E-42	0.779509	4.377628	0.0041	0.006393
1	109804646	rs4970833	4.20E-11	0.36963	5.983847	0.003687	0.002726
1	109810981	rs17035630	0.0066	0.36963	1.384568	0.001954	0.001444
1	109810544	rs2281894	7.20E-05	0.36963	1.71544	0.001058	0.000782
1	109807099	rs6689614	4.60E-11	0.36963	2.897931	0.000706	0.000522

1. GWAS_P: the original GWAS *P*-value; 2. the regulatory probability for the condition-dependent model using HepG2 epigenomes; 3. BF: the Bayes factor of the composite model; 4. Composite_P: the probability for the composite model; 5: the posterior probability of the full model.

Table S11: Functional evidence of the top five SNPs using the HepG2 epigenome.

Chr	Pos	SNP_ID	GWAS Trait | The most significant P-value | PMID (From GWASdb[1])	eQTL tissue/cell | Associated gene | The most significant P-value | PMID	TF motif | Alleles | Log(P-value for binding affinity) change (From GWAS3D[2])
1	109826136	rs657420	LDL cholesterol | 3.94E-16 | 23063622, 20686566	Skin_Sun_Exposed_Lower_leg | AMIGO1 | 2.51E-08 | 25954001<br>Skin_Not_Sun_Exposed_Suprapubic | AMIGO1 | 2.59E-08 | 25954001<br>Adipose_Subcutaneous | AMIGO1 | 5.29E-08 | 25954001<br>Cells_Transformed_fibroblasts | AMIGO1 | 2.17E-07 | 25954001<br>Nerve_Tibial | AMIGO1 | 5.05E-07 | 25954001<br>Muscle_Skeletal | AMIGO1 | 4.13E-06 | 25954001<br>Nerve_Tibial | SYPL2 | 1.15E-06 | 25954001<br>Thyroid | AMIGO1 | 1.20E-06 | 25954001<br>Whole_Blood | SORT1 | 2.92E-06 | 24013639<br>Thyroid | PSRC1 | 1.09E-05 | 25954001	YY1_known2 | C/T | -3.219181 -> -8.108031<br>E2F_known5 | C/T | -6.719181 -> -3.765598<br>HNF4_known2 | C/T | -7.217416 -> -4.38194<br>E2F_known2 | C/T | -6.695662 -> -3.914967<br>ZBTB33_known1 | C/T | -4.644391 -> -7.130899
1	109817838	rs660240	LDL cholesterol | 8.30E-41 | 20686566, 23063622, 18262040<br>Coronary heart disease | 1.00E-26 | 20864672<br>Cardiovascular disease risk factors| 2.00E-22 | 21943158<br>Lipoprotein-associated phospholipase A2 mass | 7.39E-17 | 22003152<br>Metabolite levels | 2.38E-12 | 22286219	Muscle_Skeletal | CELSR2 | 1.60E-32 | 25954001<br>Liver | SORT1 | 3.33E-19 | 25954001<br>Liver | PSRC1 | 3.78E-14 | 25954001<br>Liver | CELSR2 | 3.17E-13 | 25954001<br>Esophagus_Mucosa | PSRC1 | 4.25E-10 | 25954001<br>Testis | PSRC1 | 3.79E-08 | 25954001<br>Pancreas | PSRC1 | 2.83E-07 | 25954001<br>Whole_Blood | PSRC1 | 4.52E-07 | 25954001<br>Brain_Cortex | PSRC1 | 1.93E-06 | 25954001	BRCA1_known2 | T/C | -14.436087 -> -10.375644<br>E2F_known8 | T/C | -4.207875 -> -6.884375<br>GR_known6 | T/C | -5.630754 -> -3.0<br>GATA_known8 | T/C | -6.330637 -> -3.892992<br>E2F_known2 | T/C | -3.238518 -> -5.650919
1	109818530	rs646776	LDL cholesterol | 7.90E-74 | 23063622, 18193044, 18262040, 20686566, 19060910<br>Lipid levels | 1.77E-53 | 19936222, 19060911, 19802338, 19913121, 23236364<br>Apolipoprotein B levels | 3.90E-41 | 23100282<br>Progranulin levels | 2.00E-30 | 21087763<br>Multiple cancers | 6.10E-28 | 23103227<br>Lipoprotein-associated phospholipase A2 activity and mass | 2.15E-17 22003152 | 22003152<br>Metabolite levels | 2.65E-12 | 22286219<br>Coronary heart disease | 7.90E-12| 21966275, 21347282, 21378988<br>Myocardial infarction | 8.00E-12 | 19198609<br>Cardiovascular disease risk factors | 9.37E-9 | 21943158	Muscle_Skeletal | CELSR2 | 1.06E-34 | 25954001<br>Liver | CELSR2 | 3.09E-24 | 18462017<br>Liver | SORT1 | 3.44E-20 | 25954001<br>Liver | PSRC1 | 1.57E-15 | 25954001<br>Liver | CELSR2 | 5.44E-15 | 25954001<br>Esophagus_Mucosa | PSRC1 | 1.24E-11 | 25954001<br>Whole_Blood | PSRC1 | 1.98E-07 | 25954001<br>Liver | PSMA5 | 2.44E-07 | 18462017<br>Pancreas | PSRC1 | 3.49E-07 | 25954001<br>Testis | PSRC1 | 5.60E-07 | 25954001<br>Brain_Cortex | PSRC1 | 8.68E-07 | 25954001<br>Skin_Not_Sun_Exposed_Suprapubic | PSRC1 | 2.73E-06 | 25954001	GATA_known14 | C/T | -4.369146 -> -6.877495<br>Myc_known8 | C/T | -5.789017 -> -3.305023<br>GATA_known10 | C/T | -3.462016 -> -5.929344<br>GR_known2 | C/T | -5.291566 -> -3.0<br>YY1_known3 | C/T | -6.471606 -> -4.217397
1	109818306	rs629301	LDL cholesterol | 5.00E-241 | 24097068, 20686565, 23063622, 18262040, 20686566, 21541012, 19913121<br>Lipoprotein-associated phospholipase A2 activity and mass | 1.78E-17 | 22003152<br>Metabolite levels | 2.62E-12 | 22286219	Muscle_Skeletal | CELSR2 | 1.06E-34 | 25954001<br>Liver | SORT1 | 3.44E-20 | 25954001<br>Liver | PSRC1 | 1.57E-15 | 25954001<br>Liver | CELSR2 | 5.44E-15 | 25954001<br>Esophagus_Mucosa | PSRC1 | 1.24E-11 | 25954001<br>Whole_Blood | PSRC1 | 1.98E-07 | 25954001<br>Pancreas | PSRC1 | 3.49E-07 | 25954001<br>Testis | PSRC1 | 5.60E-07 | 25954001<br>Brain_Cortex | PSRC1 | 8.68E-07 | 25954001<br>Skin_Not_Sun_Exposed_Suprapubic | PSRC1 | 2.73E-06 | 25954001	YY1_known6 | G/T | -7.699671 -> -12.724866<br>YY1_known2 | G/T | -6.454206 -> -11.0021<br>Mef2_known2 | G/T | -11.781189 -> -8.729368<br>YY1_known5 | G/T | -6.376764 -> -9.09393<br>Pou2f2_known5 | G/T | -3.967087 -> -6.46091
1	109817590	rs12740374	LDL cholesterol | 3.75E-69 | 23063622, 22629316, 19060906, 20686566, 23236364, 18262040, 21541012, 19913121<br>Lipoprotein-associated phospholipase A2 activity and mass | 2.00E-22 | 23118302, 22003152<br>Metabolic syndrome phenotype | 8.00E-16 | 22022282<br>Metabolite levels | 1.24E-11 | 22286219	Muscle_Skeletal | CELSR2 | 1.16E-35 | 25954001<br>Liver | SORT1 | 4.01E-27 | 25954001<br>Liver | CELSR2 | 5.16E-19 | 25954001<br>Liver | PSRC1 | 5.64E-19 | 25954001<br>Esophagus_Mucosa | PSRC1 | 5.59E-12 | 25954001<br>Pancreas | PSRC1 | 7.16E-07 | 25954001<br>Whole_Blood | PSRC1 | 2.07E-06 | 25954001<br>Testis | PSRC1 | 5.69E-06 | 25954001<br>Esophagus_Mucosa | CELSR2 | 4.98E-06 | 25954001<br>Skin_Sun_Exposed_Lower_leg | CELSR2 | 7.33E-06 | 25954001	CEBPB_known1 | G/T | -8.132542 -> -14.392124<br>CEBPB_known3 | G/T | -6.464692 -> -12.172171<br>CEBPB_known5 | G/T | -9.26345 -> -14.369396<br>CEBPB_known6 | G/T | -6.525939 -> -10.785877<br>BRCA1_known2 | G/T | -3.57882 -> -6.866675

[1] Li MJ, *et al*. 2016. GWASdb v2: an update database for human genetic variants identified by genome-wide association studies. ***Nucleic Acids Res***.
[2] Li MJ, *et al*. 2013. GWAS3D: Detecting human regulatory variants by integrative analysis of genome-wide associations, chromosome interactions and histone modifications. ***Nucleic Acids Res.***

Table S12: Potential novel RA regulatory SNPs with moderate significant P-values. These candidates were prioritized using blood Mo-CD14+ epigenomic marks (Top 50).

**SNP**	**Combined_P***	**GWAS P-value**	**SNP**	**Combined_P***	**GWAS P-value**
rs874628	0.506966	0.00036	rs695871	0.303774	0.0003
rs1933437	0.453914	0.00023	rs2076615	0.29915	0.00022
rs1050069	0.452307	0.00016	rs55960411	0.293754	0.00056
rs149271	0.445721	0.00097	rs752118	0.293195	9.00E-05
rs1126931	0.414567	0.00052	rs61776582	0.292749	0.00056
rs34891485	0.398838	0.00014	rs4845635	0.290597	0.00019
rs7097397	0.3932	0.00039	rs1453559	0.286038	6.60E-08
rs17227424	0.389769	0.00085	rs8011558	0.284322	4.40E-06
rs74929644	0.373818	0.00046	rs16891427	0.280171	1.10E-05
rs1800686	0.366551	9.00E-05	rs7198606	0.279276	0.00053
rs118054983	0.34874	3.60E-05	rs3757386	0.273307	0.0004
rs3825568	0.343718	3.70E-06	rs141333506	0.270571	0.0007
rs113892147	0.342055	7.60E-05	rs4657055	0.269013	0.00077
rs12889006	0.341814	4.00E-06	rs79858408	0.265983	0.00044
rs33959228	0.335268	0.00016	rs1465788	0.265486	3.50E-06
rs17751061	0.324599	0.00011	rs117820542	0.264112	0.00045
rs710845	0.322226	0.00084	rs187060419	0.262165	0.00047
rs4808970	0.318964	6.30E-05	rs73740408	0.262047	0.00023
rs3746435	0.315127	0.00053	rs6573857	0.259303	3.60E-06
rs2070179	0.313192	0.00093	rs470082	0.258763	3.60E-06
rs113233347	0.311692	0.0008	rs6990813	0.256012	0.00033
rs174538	0.310173	0.00025	rs6060266	0.255235	0.00091
rs10747783	0.30991	9.10E-06	rs2072052	0.254986	1.60E-05
rs874881	0.307804	0.00016	rs34480360	0.253426	4.20E-06
rs10849962	0.304733	0.00083	rs138913261	0.252925	0.00016

*Combined_P: the posterior probability of the full model.

Table S13: The ratio of effective number of independent markers on each chromosome. In most chromosomes, the ratios are larger than original GWAS signals, indicating that context-dependent model improved the discovery of GWAS SNPs by identifying more LD-independent signals.

CELLS (%)	chr1	chr2	chr3	chr4	chr5	chr6	chr7	chr8	chr9	chr10	chr11	chr12	chr13	chr14	chr15	chr16	chr17	chr18	chr19	chr20	chr21	chr22	chrX
**BLD.DND41**	0.32	0.3	0.16	0.07	0.1	0.32	0.11	0.15	0.18	0.39	0.13	0.15	0	0.23	0.15	0.08	0.1	0.12	0.2	0	1.02	0.21	0.76
**BLD.GM12878**	0.31	0.27	0.21	0.06	0.09	0.47	0.22	0.09	0.16	0.4	0.11	0.12	0	0.26	0.13	0.07	0.13	0.22	0.12	0.09	1.11	0.31	0.62
**CRVX.HeLa-S3**	0.31	0.25	0.15	0	0.09	0.35	0.13	0.06	0.12	0.23	0.04	0.1	0	0.17	0	0.08	0.1	0	0.14	0	0.72	0.11	0.48
**LIV.HepG2**	0.28	0.21	0.15	0	0.05	0.22	0.21	0.13	0.11	0.26	0.12	0.09	0	0.26	0.07	0.06	0.12	0.12	0.16	0.05	0.96	0.27	0.76
**BRST.HMEC**	0.26	0.17	0.07	0.11	0	0.26	0.15	0.05	0.13	0.33	0.13	0.1	0	0.19	0.06	0.08	0.1	0.1	0.13	0.07	0.53	0.32	0.46
**MUS.HSMM**	0.27	0.24	0.15	0	0.04	0.29	0.09	0.05	0.11	0.37	0.09	0.14	0.09	0.19	0.06	0.09	0.09	0.1	0.1	0	0.52	0.1	0.5
**VAS.HUVEC**	0.28	0.19	0.15	0	0.08	0.3	0.14	0	0.12	0.3	0.08	0.13	0.09	0.19	0	0.08	0.1	0.1	0.2	0.08	0.36	0.16	0.49
**BLD.K562**	0.31	0.3	0.17	0.13	0.15	0.32	0.16	0.12	0.11	0.22	0.08	0.12	0	0.24	0.07	0.08	0.11	0.24	0.2	0.08	0.69	0.23	0.64
**BLD.Mo-CD14+**	0.31	0.2	0.22	0	0.04	0.34	0.22	0.13	0.1	0.2	0.12	0.16	0	0.24	0.14	0.08	0.13	0.12	0.23	0.07	0.35	0.14	0.62
**BRN.NH-A**	0.29	0.21	0.16	0.05	0.04	0.25	0.12	0.05	0.06	0.43	0.1	0.14	0.09	0.19	0.13	0.08	0.11	0.1	0.14	0.08	0.37	0.15	0.49
**SKIN.NHDF-Ad**	0.26	0.29	0.14	0.11	0.08	0.31	0.09	0.05	0.11	0.39	0.08	0.11	0	0.25	0	0.08	0.09	0.1	0.15	0	0.7	0.16	0.55
**LNG.NHLF**	0.23	0.26	0.15	0.05	0.04	0.26	0.15	0.05	0.07	0.4	0.12	0.09	0	0.26	0.12	0.07	0.11	0.11	0.18	0.07	0.36	0.14	0.55
**BONE.OSTEO**	0.33	0.22	0.12	0	0.04	0.29	0.19	0.11	0.1	0.33	0.13	0.04	0	0.21	0	0.09	0.11	0.11	0.17	0.07	0.74	0.1	0.7
**composite**	0.25	0.27	0.12	0	0	0.22	0.12	0.11	0.14	0.31	0.13	0.07	0.1	0.24	0.06	0.07	0.13	0.1	0.13	0.07	0.54	0.14	0.43
**GWAS**	0.09	0.06	0.02	0.02	0.03	0.08	0.02	0.02	0.02	0.04	0.03	0.02	0.02	0.01	0.05	0.02	0.03	0.04	0.05	0.02	0.1	0.04	0.03

Note: ‘0’ means no SNPs on the particular chromosome satisfied rheumatoid arthritis GWAS $P < 1 \times 10^{-5}$ after prioritization.

Table S14: Results of the pathway enrichment analysis (-Log10FDR) using improved genes. Weighting by blood cell lines (especially Mo-CD14+ and GM12878) show higher enrichment in immune system pathways than weighting by other tissues/cell types.

CELLS	IMMUNE SYSTEM	IMMUNOREGULATORY INTERACTIONS	INNATE IMMUNE SYSTEM	ADAPTIVE IMMUNE SYSTEM	CYTOKINE SIGNALING IN IMMUNE SYSTEM
BLD Mo-CD14+	6.43	1.99	0	2.4	4.18
BLD GM12878	4.91	0	1.4	0	2.51
BLD DND41	4.6	1.71	0	1.71	1.4
BLD K562	3.86	0	0	0	1.79
LIV HepG2	3.56	0	0	0	1.6
BONE OSTEO	3.26	0	0	0	3.39
BRN NH-A	2.92	0	0	0	2.89
LNG NHLF	2.27	0	0	0	0
VAS HUVEC	2.15	0	0	0	2.35
CRVX HeLa-S3	1.84	0	0	0	0
BRST HMEC	1.63	0	0	0	0
MUS HSMM	0	0	0	0	1.78
SKIN NHDF-Ad	0	0	0	0	0

Note: ‘0’ means the relevant pathway is not significantly enriched (FDR > 0.05) in the analysis.

Abbreviations: BLD - Blood; LNG - Lung; SKIN - Skin; BONE - Bone; LIV - Liver; VAS - Vascular; MUS - Muscle; BRN - Brain; CRVX - Cervix; BRST – Breast.
